# HMGA1 amplifies Wnt signalling and expands the intestinal stem cell compartment and Paneth cell niche

**DOI:** 10.1038/ncomms15008

**Published:** 2017-04-28

**Authors:** Lingling Xian, Dan Georgess, Tait Huso, Leslie Cope, Amy Belton, Yu-Ting Chang, Wenyong Kuang, Qihua Gu, Xiaoyan Zhang, Stefania Senger, Alessio Fasano, David L. Huso, Andrew J. Ewald, Linda M. S. Resar

**Affiliations:** 1 Division of Hematology, Department of Medicine, The Johns Hopkins University School of Medicine, 720 Rutland Avenue, Ross Research Building, Room 1025, Baltimore, Maryland 21205, USA; 2 Department of Cell Biology, The Johns Hopkins University School of Medicine, 855 North Wolfe Street, Baltimore, Maryland 21205, USA; 3 Division of Biostatistics, Department of Oncology, The Johns Hopkins University School of Medicine, 550 North Broadway, Baltimore, Maryland 21205, USA; 4 Department of Pathology, Pathobiology Graduate Program, The Johns Hopkins University School of Medicine, 720 Rutland Avenue, Ross Research Building, Room 1025, Baltimore, Maryland 21205, USA; 5 Department of Pediatrics, Mucosal Immunology and Biology Research Center, Harvard Medical School, Massachusetts General Hospital East, 16th Street, Building 114, Charlestown, Massachusetts 02114, USA; 6 Department of Molecular and Comparative Pathobiology, The Johns Hopkins University School of Medicine, Baltimore, Maryland 21205, USA; 7 Department of Oncology, The Johns Hopkins University School of Medicine, Baltimore, Maryland 21205, USA; 8 Department of Pathology and Institute for Cellular Engineering, The Johns Hopkins University School of Medicine, Baltimore, Maryland 21205, USA

## Abstract

High-mobility group A1 (Hmga1) chromatin remodelling proteins are enriched in intestinal stem cells (ISCs), although their function in this setting was unknown. Prior studies showed that Hmga1 drives hyperproliferation, aberrant crypt formation and polyposis in transgenic mice. Here we demonstrate that Hmga1 amplifies Wnt/β-catenin signalling to enhance self-renewal and expand the ISC compartment. Hmga1 upregulates genes encoding both Wnt agonist receptors and downstream Wnt effectors. Hmga1 also helps to ‘build' an ISC niche by expanding the Paneth cell compartment and directly inducing *Sox9*, which is required for Paneth cell differentiation. In human intestine, *HMGA1* and *SOX9* are positively correlated, and both become upregulated in colorectal cancer. Our results define a unique role for Hmga1 in intestinal homeostasis by maintaining the stem cell pool and fostering terminal differentiation to establish an epithelial stem cell niche. This work also suggests that deregulated *Hmga1* perturbs this equilibrium during intestinal carcinogenesis.

## Abstract (web summary)

The function of high mobility group A1 (Hmga1) chromatin remodelling proteins in intestinal stem cells (ISC) is unknown. Here, the authors show that Hmga1 amplifies Wnt/β-catenin signalling to enhance self-renewal and induces *Sox9* to expand the Paneth cell compartment and enrich the ISC niche.

Intestinal stem cells (ISCs) provide a paradigm for studying adult stem cell function due to their exceptional self-renewal potential and repetitive structural organization^1^,^2^,^3^,^4^,^5^. Indeed, the intestinal lining is among the most highly regenerative tissues, renewing itself every 3–5 days to protect the gut from pathogens and maintain nutrient intake essential for life. Over the past decade, a population of self-renewing, columnar epithelial cells located at the base of the intestinal crypts has been identified and characterized as ISCs^1^,^2^,^3^,^4^,^5^. They are marked by the serpentine receptor, leucine-rich repeat containing G-protein-coupled receptor 5 (Lgr5), which mediates Wnt signalling cues from the niche^5^. Lineage tracing experiments demonstrate that these ISCs are responsible for the exuberant regeneration and tissue homeostasis in intestinal epithelium^1^,^4^,^6^. Despite extensive study, the molecular mechanisms that govern their behaviour are only beginning to be elucidated^1^,^2^,^3^,^4^,^5^,^6^,^7^,^8^,^9^. Previous work also demonstrates that aberrant expression or mutation of key regulators of ISCs leads to neoplastic growth and intestinal carcinogenesis^10^,^11^.

Emerging evidence highlights the central role for chromatin structure and chromatin-binding proteins in maintaining stem cell properties. In fact, recent work found that the high-mobility group A1 chromatin remodelling proteins (HMGA1, formerly HMG-I/Y) regulate stem cell properties in cancer^12^,^13^,^14^,^15^,^16^,^17^,^18^, although their role in normal development has remained elusive. The *HMGA1* gene encodes the HMGA1a and HMGA1b isoforms^19^,^20^,^21^, which function as architectural transcription factors. HMGA1 proteins bind to specific DNA sequences^13^,^22^,^23^,^24^, modulate chromatin structure^23^ and recruit other transcriptional complexes to regulatory regions throughout the genome^13^,^22^,^23^. *HMGA1* is highly expressed during embryogenesis, with high levels in normal embryonic stem cells^13^,^16^,^25^,^26^. Postnatally, *HMGA1* is expressed in adult stem cells, such as hematopoietic^27^,^28^ and intestinal stem cells^29^, but absent or barely detectable in mature, differentiated tissues. In cancer, *HMGA1* becomes aberrantly expressed through oncogenic transcription factors and epigenetic alterations, or in rare cases, chromosomal translocation events^13^,^17^,^30^,^31^. Moreover, *HMGA1* is overexpressed in most high-grade or poorly differentiated cancers studied to date, and high levels portend a poor prognosis in diverse tumours^12^,^13^,^14^,^15^,^16^,^17^,^18^,^26^,^31^,^32^,^33^,^34^,^35^,^36^. In murine tumour xenografts, *HMGA1* drives tumour progression and cancer stem cell properties, at least in part, by inducing stem cell transcriptional networks^12^,^13^,^14^,^15^,^16^,^17^,^18^. In human embryonic stem cells, HMGA1 maintains a de-differentiated state by upregulating genes involved in stemness and pluripotency^16^. Moreover, HMGA1 is required for reprogramming somatic cells to induced pluripotent stem cells by the Yamanaka factors; disrupting *HMGA1* expression or function prevents the derivation of fully reprogrammed cells^16^. Given its dual role in normal development and cancer, further studies to dissect *HMGA1* function in each setting are needed to determine the therapeutic potential of targeting *HMGA1* in cancer or harnessing its function for tissue regeneration.

We previously demonstrated that transgenic mice overexpressing murine *Hmga1* from the H-2K^b^ promoter and immunoglobulin μ enhancer all succumb to lymphoid tumours^35^; females also develop uterine sarcomas^36^. In this model, the transgene is expressed in the intestines^14^ in addition to lymphoid cells^35^ and uterine tissue^36^. The *Hmga1* transgenics develop marked proliferative changes in the epithelium of the small and large intestine, with aberrant crypt formation and polyposis^14^. To determine how Hmga1 disrupts tissue homeostasis in the intestines of transgenic mice and intestinal cancers overexpressing *HMGA1*, we examined its expression and function in our transgenic model and in intestinal organoids. We discover that *Hmga1* expands the ISC pool and Paneth cell niche *in vivo.* Hmga1 is a key factor involved in the organization of ISCs into three-dimensional (3D) organoids *in vitro*. We also find that *Hmga1* enhances ISC expansion and self-renewal by amplifying Wnt/β-catenin signalling. Hmga1 also directly upregulates *Sox9* and expands the Paneth cell niche. This is an example of Hmga1 fostering terminal differentiation to establish a stem cell niche. Moreover, both *HMGA1* and *SOX9* are positively correlated in human intestinal epithelium, and both become markedly upregulated in colorectal cancer. These results reveal a unique role for *Hmga1* in maintaining both the ISC pool and niche cells within intestinal crypts and suggest that this equilibrium is perturbed when *Hmga1* becomes deregulated during carcinogenesis.

## Results

### Hmga1 drives expansion of the ISC compartment

**Figure 1:**
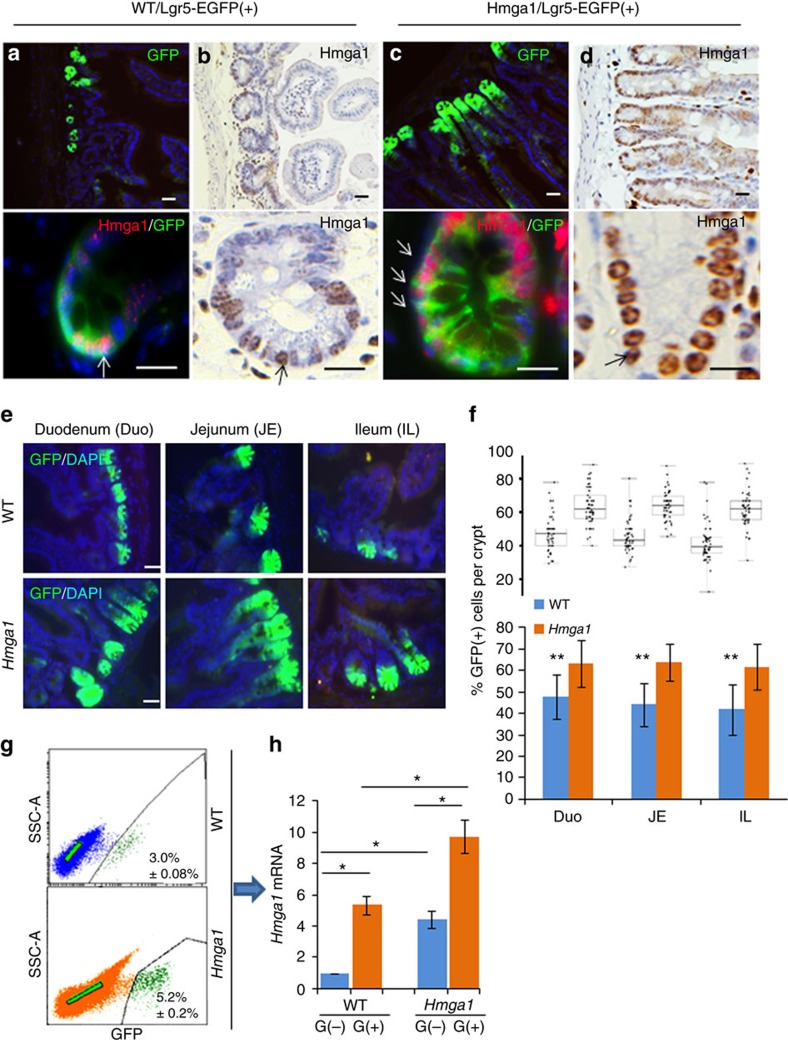
Hmga1 localizes to ISCs and its overexpression expands the ISC compartment in transgenic mice. (**a**) ISCs from WT/Lgr5-EGFP+ mice stain green (upper panel) in small intestinal cross-sections. Hmga1 stains red and localizes to GFP+ ISCs (lower panel). (**b**) Intranuclear Hmga1 stains brown (immunohistochemistry (IHC)) in small intestinal cross-sections from WT/Lgr5-EGFP+ mice. (**c**) ISCs stain green in Hmga1/Lgr5-EGFP+ transgenic mice and extend further up the base of the crypt in *Hmga1* transgenic mice compared to WT mice. (Cells with red nuclear staining outside of crypts are *Hmga1* transgenic lymphocytes) (**d**) Intranuclear Hmga1 stains brown (IHC) in small intestinal cross-sections from Hmga1/Lgr5-EGFP+ mice. (**e**) Fluorescent staining identifies GFP+ ISCs at each region in the small intestine of WT and *Hmga1* mice (duodenum, jejunum, ileum). (**f**) The percentage (mean±s.d.) of GFP+ ISCs per crypt in WT and *Hmga1* transgenic mice are shown; **P<0.00001, Mann–Whitney test (*n*=50 crypts per region, 3 mice per genotype). Dot plot shows individual data points. (**g**) *Hmga1* transgenic mice have higher GFP+ ISC frequency as assessed by flow cytometry; mean frequency±s.d. from two experiments are shown. (**h**) *Hmga1* mRNA is enriched in ISCs isolated by flow cytometry for GFP+ cells using quantitative, real-time PCR (qPCR) in WT and *Hmga1* transgenic mice. *Hmga1* is higher in both GFP+ ISCs and GFP− crypt cells from the *Hmga1* transgenic model compared to WT mice. Bars show mean relative *Hmga1* expression±s.d. from three experiments performed in triplicate; *Gapdh* was used as a loading control; *P<0.05; two-tailed Student's *t*-test. Scale bars, 20 μm.

A prior gene expression profile study showed that *Hmga1* is among the genes enriched in Lgr5+ ISCs (ref. 29). *HMGA1* is also among the genes most highly expressed in diverse epithelial human cancers as compared to normal epithelium, including intestinal malignancies^12^,^13^,^14^,^17^,^33^. We therefore sought to elucidate the functional role of Hmga1 in ISCs, both in normal intestinal epithelial homeostasis and in intestinal neoplasia. To this end, we crossed our *Hmga1* transgenic mice onto Lgr5-EGFP mice^6^, which mark Lgr5+ ISCs with enhanced green fluorescent protein (EGFP). The *Hmga1* transgene is driven by the H-2K^b^ promoter and μ enhancer, which confer transgene expression in intestinal crypt basilar cells^37^, lymphoid cells^35^ and uterine tissue^36^. In both *Hmga1* transgenic and wild-type (WT) mice, Hmga1 protein localizes to the nuclei of Lgr5+ ISCs (Fig. 1a–d). Interestingly, Lgr5+ ISCs extend further up the crypts in the *Hmga1* transgenic mice compared to WT mice in all regions of the small intestine (duodenum, jejunum, ileum; Fig. 1a–f), consistent with the expansion in the ISC pool and enhanced self-renewal *in vivo*. The percentage of Lgr5+ ISCs per total crypt cells by fluorescent stain (Fig. 1f; **P<0.00001; Mann-Whitney test) and relative frequency of Lgr5+ISCs per crypt cell isolates by fluorescence-activated cell sorting (FACS) were significantly (*P*<0.05; two-tailed Student's *t*-test) increased in the *Hmga1* transgenic small intestine (Fig. 1g,h), further validating the expansion in ISCs. To determine whether *Hmga1* gene expression accounts for the elevated protein levels in the Lgr5+ ISCs, we assessed *Hmga1* mRNA in WT mice and found that it is increased by ∼4-fold in Lgr5+ ISCs isolated by FACS compared to Lgr5− cells (Fig. 1g,h). We also assessed *Hmga1* mRNA in crypt cells from the transgenic model and found that *Hmga1* expression is increased about twofold in both Lgr5+ ISCs and Lgr5− cells compared to the same cell populations in WT mice. In both WT and transgenic models, *Hmga1* mRNA is enriched in the Lgr5+ ISC population (Fig. 1g,h).

**Figure 2:**
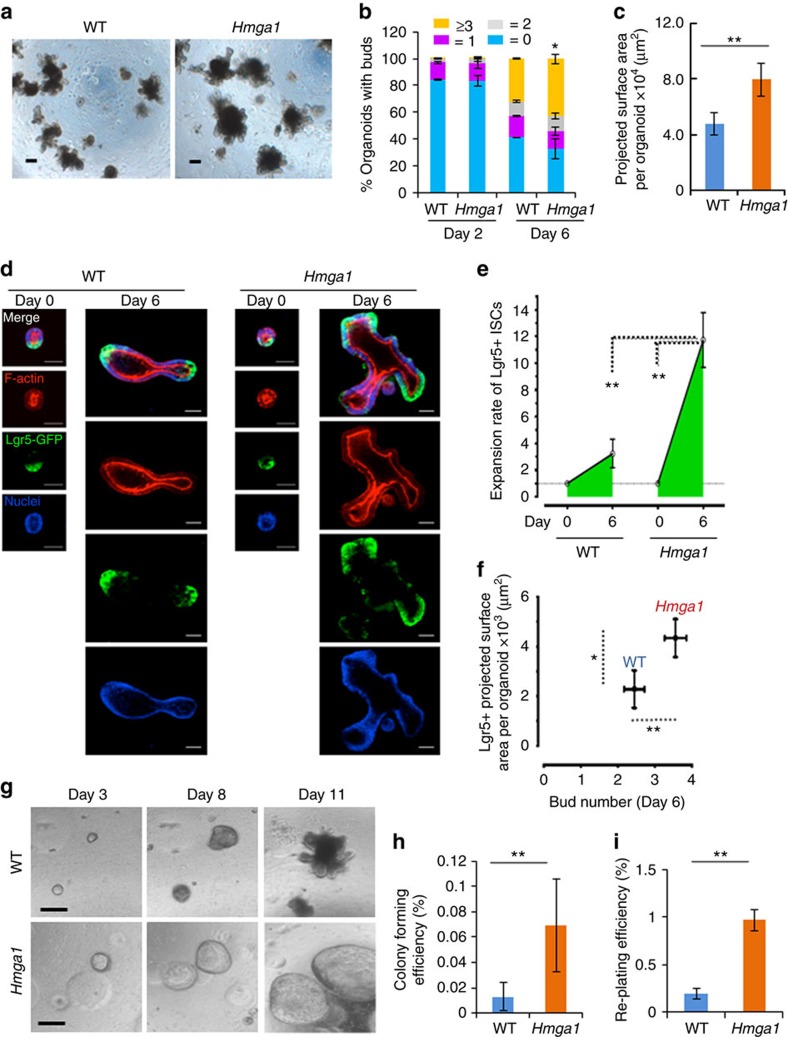
Hmga1 enhances ISC function in gut organoid cultures. (**a**) Typical 3D organoids cultured from crypts are shown from WT or *Hmga1* transgenic mice. (**b**) Bud numbers were ascertained in 3D organoids from isolated crypts (*n*>100 organoids per mouse; 3 mice per genotype). Bars show mean percentage of organoids±s.d. with different bud numbers. (Yellow≥3, grey=2, pink=1, blue=0). The mean percentage of organoids with ≥ 3 buds was increased in *Hmga1* organoids compared to WT organoids. *P<0.05; two-tailed Student's *t*-test. (**c**) Organoid projected surface area (PSA) is shown (mean±s.d.) from WT or *Hmga1* mice (*n*>20 organoids/genotype). **P<0.01; two-tailed Student's *t*-test. (**d**) Representative confocal imaging of crypt cells (day 0) and organoids (day 6) with GFP+ staining for Lgr5+ ISCs, Phalloidin (F-actin; red) for cell clusters and organoid structure, and Hoechst (blue) for nuclei are shown. (**e**) Relative expansion rate of ISCs was calculated by the ratio of the PSA of GFP+ ISCs on day 6 (*n*≥42 organoids per group) over the PSA on day 0 (*n*≥72 crypt cell clusters/group). **P<0.01; Mann–Whitney test. (**f**) ISC PSA (*y* axis) and bud number (*x* axis) at days 0 and 6 are shown. **P<0.01, *P<0.05; Mann–Whitney test. (**g**) Lgr5-GFP+ ISCs were purified by flow cytometry and cultured in matrigel. (**h**) Colony (organoid)-forming efficiency (mean±s.d.) was calculated from purified GFP+ ISCs isolated as single cells (*n*=3,000 cells per group) from WT and *Hmga1* mice in three experiments; **P<0.01; two-tailed Student's *t*-test. (**i**) Re-plating efficiency (mean±s.d.; *n*=3,000 cells per group) was assessed after dissociating colonies from purified GFP+ ISCs isolated as single cells from WT and *Hmga1* mice using flow cytometry. **P<0.01; two-tailed Student's *t*-test. Scale bars, 50 μm.

ISCs are regulated by factors from the stromal compartment in addition to intestinal epithelial cells^1^,^2^,^3^,^4^,^5^,^6^,^7^,^8^,^9^,^38^,^39^,^40^. To define the role of Hmga1 in ISCs within the epithelial compartment, we used organoids, an *in vitro* intestinal crypt cell culture model^1^,^2^,^3^,^4^,^41^. Organoid buds are a surrogate for ISC function because they comprise crypt-like structures with ISCs on the tips; differentiated epithelial cells extend towards the luminal centres of the organoids. We derived organoids from small intestinal epithelial crypt cells isolated from transgenic or WT mice and compared bud formation and projected surface area. Prior to culture, an equal number of crypts from WT or *Hmga1* transgenic mice of similar sizes were isolated by passage through a 70 μM filter^41^. Similar to the histologic results in the transgenic mouse intestine, we found a marked increase in projected surface area per organoid (Image-Pro Plus Version 6) and bud number per organoid in those derived from the *Hmga1* transgenic mice as compared to WT controls (Fig. 2a–c; Supplementary Fig. 1a,b). There were more *Hmga1* organoids with >3 buds as compared to WT organoids by day 6 in culture (Fig. 2b). The majority of WT organoids had ⩽1 buds per organoid, while the majority of *Hmga1* organoids had ≥2 buds at day 6 (Fig. 2b). Next, we isolated small crypts by passage through a 40 μM filter^40^,^41^ and performed time-lapsed confocal microscopy^42^,^43^,^44^,^45^ to ascertain the rate of self-renewal in Lgr5+ ISCs and bud development from day 0 to day 6 in 3D organoid culture (Fig. 2d–f). A representative image of crypt cells from days 0 and 6 from WT mice or *Hmga1* transgenics, respectively, are shown (Fig. 2d). We discovered a marked increase in the rate of self-renewal at day 6 in the Lgr5+ ISCs from the *Hmga1* transgenics compared to the WT mice based on the projected surface area of Lgr5+ cells (μManager software; Stanford Photonics; Fig. 2e,f). Similar to our prior results using standard microscopy, we also found more buds and greater projected surface area of Lgr5+ cells in the *Hmga1* organoids versus the WT organoids by confocal microscopy (Fig. 2f).

Previous work from our group demonstrated that forced expression of *Hmga1* prevents differentiation in human embryonic stem cells^16^. We therefore reasoned that *Hmga1* expression would predominate at the crypt-like regions enriched for ISCs at the bud tips and decrease in regions of differentiated cells at the base of the buds. To test this, we assessed Hmga1 protein levels throughout the organoids and found that Hmga1 is enriched at the bud tips where ISCs predominate, and undetectable in the differentiated cells, suggesting that differentiation is permitted in cells with lower levels of Hmga1 (Supplementary Fig. 2). Together, these results suggest that Hmga1 is a key factor for ISC maintenance and function.

Because we could not exclude the possibility that the enhanced stem cell function observed in the *Hmga1* organoids resulted from *in vivo* exposure to lymphoid or other cells with transgenic *Hmga1* expression and downstream factors, we engineered WT organoids to overexpress *Hmga1.* To this end, we transduced organoids via lentivirus expressing *Hmga1* and GFP (FUGW-Hmga1) and compared this to control organoids transduced with lentivirus expressing GFP alone (FUGW). The WT organoids engineered to overexpress *Hmga1* exhibited a similar phenotype to the organoids derived from the *Hmga1* mice with increased bud formation (Supplementary Fig. 3), demonstrating that this phenotype was dependent on *Hmga1*.

To determine whether the enhanced stem cell function in organoid-forming ability and bud development resulted from cell-autonomous properties of the *Hmga1* ISCs or from ISC interactions with other crypt epithelial cells, we purified Lgr5+ ISCs isolated as single cells from transgenic and WT mice using flow cytometry and compared their efficiency in organoid formation and re-plating assays. The purified *Hmga1* ISCs generated organoids at a fivefold greater efficiency as compared to WT ISCs (Fig. 2g,h). Moreover, re-plating efficiency was also enhanced fivefold in the *Hmga1* ISCs (Fig. 2i). Interestingly, the *Hmga1* organoids generated from purified Lgr5+ ISCs adopt a cyst-like, spherical structure similar to the morphology observed in organoids treated with Wnt or engineered to overexpress *Ascl2*, a transcription factor important in maintaining stem cell identity in ISCs^7^. To determine whether *Hmga1* organoids remained in a cyst-like structure because they were comprised predominantly of undifferentiated Lgr5+ stem cells, we stained for the ISC marker, Lgr5-GFP+. Strikingly, most of the cells within the *Hmga1* organoids generated from purified *Hmga1* Lgr5-GFP+ ISCs maintain this stem cell marker (Lgr5-GFP+; Supplementary Fig. 4), in contrast to WT organoids, which only stain for Lgr5-GFP+ at the tips of the buds (Fig. 2d). Together, these studies demonstrate that *Hmga1* maintains undifferentiated Lgr5+ ISCs and enhances self-renewal in this model.

**Figure 3:**
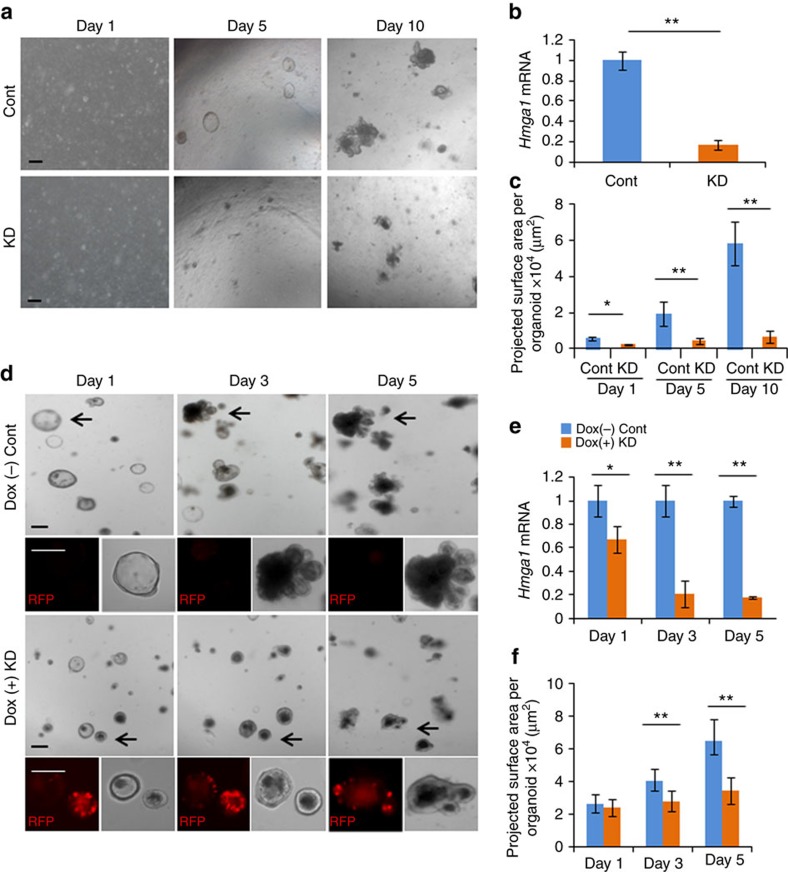
*Hmga1* deficiency disrupts ISC function in 3D organoids. (**a**) Representative images of organoid formation after silencing *Hmga1* by delivery of lentiviral vector expressing shRNA targeting *Hmga1* (knockdown or KD) or control lentivirus (Cont) are shown. Scale bars, 50 μm. (**b**) Relative *Hmga1* expression (mean±s.d.) was assessed from two experiments performed in triplicate (via qPCR) in organoids transduced with lentivirus (Cont versus KD) at day 10. *Gapdh* was used to control for loading. **P<0.01; two-tailed Student's *t*-test. (**c**) PSA (mean±s.d.) was assessed in organoids transduced with lentivirus (Cont versus KD) at the indicated time points (*n*=20 organoids per group). *P<0.05, **P<0.01; two-tailed Student's *t*-test. (**d**) Representative image of organoid formation±knockdown of *Hmga1* via an inducible lentiviral vector expressing red fluorescent protein (RFP) and shRNA targeting *Hmga1* are shown. Doxcyline (0.5 μg ml^−1^) was used to induce RFP and shRNA. Scale bars, 50 μm. (**e**) Relative *Hmga1* expression (mean±s.d.) in organoids with or without induction of lentivirus shRNA targeting *Hmga1* was assessed from two experiments performed in triplicate (via qPCR); *Gapdh* was used to control for loading. *P<0.05, **P<0.01; two-tailed Student's *t*-test. (**f**) PSA (mean±s.d.) of organoids with or without induction of *Hmga1* knockdown at the indicated time points are shown (*n*=20 organoids per group from two different virus transduction experiments). **P<0.01; Mann–Whitney test.

Next, we sought to determine whether *Hmga1* is required for self-renewal and ISC function. First, we silenced *Hmga1* in WT crypt cells using lentiviral-mediated delivery of short hairpin RNA (shRNA) targeting *Hmga1* (*shHmga1*) and compared this to WT crypt cells transduced with a control lentiviral vector in organoid cultures (Fig. 3a). Crypt cells were incubated with Wnt3a to enhance transduction efficiency as previously reported^2^,^3^. *Hmga1* expression was repressed in the organoids transduced to express the *shHmga1* (Fig. 3b). Strikingly, the crypt cells with *Hmga1* silencing formed very few organoids with smaller projected surface areas and decreased bud number, while those transduced with control vector organized into typical 3D structures and generated new buds (Fig. 3a–c; Supplementary Fig. 5a). To rule out any potential nonspecific toxicity from the *shHmga1* vector, we also tested an inducible, shRNA lentiviral vector targeting *Hmga1* (inducible-*shHmga1*), which is linked to a gene encoding red fluorescent protein (RFP); both *RFP* and *shHmga1* are induced by doxycycline. Crypt cells transduced with the lentivirus, but not induced to express the *shHmga1* vector, organized into 3D organoids with buds similar to what we observed in WT crypt cultures (Fig. 3d; top panels). As expected, the uninduced organoids (cultured without doxycycline) do not express *RFP*. In contrast, those organoids in which the *shHmga1* vector was induced by doxycycline express *RFP* and exhibit an impaired capability to proliferate and generate 3D organoids; the projected organoid surface area was decreased compared to uninduced controls (Fig. 3d,e). Bud formation was also disrupted (Supplementary Fig. 5b). *Hmga1* gene silencing in organoids transduced with the inducible *shHmga1* was confirmed by quantitative, reverse transcription PCR (qPCR; Fig. 3e). To further validate these findings, we transduced organoids derived from the *Hmga1* mice with the *shHmga1* vector and found that silencing *Hmga1* also disrupted their ability to form organoids and generate buds (Supplementary Fig. 5c). These studies indicate that *Hmga1* is a key factor involved in self-renewal, bud formation and organization into 3D organoid structures.

### Hmga1 induces self-renewal through Wnt/β-catenin signalling

**Figure 4:**
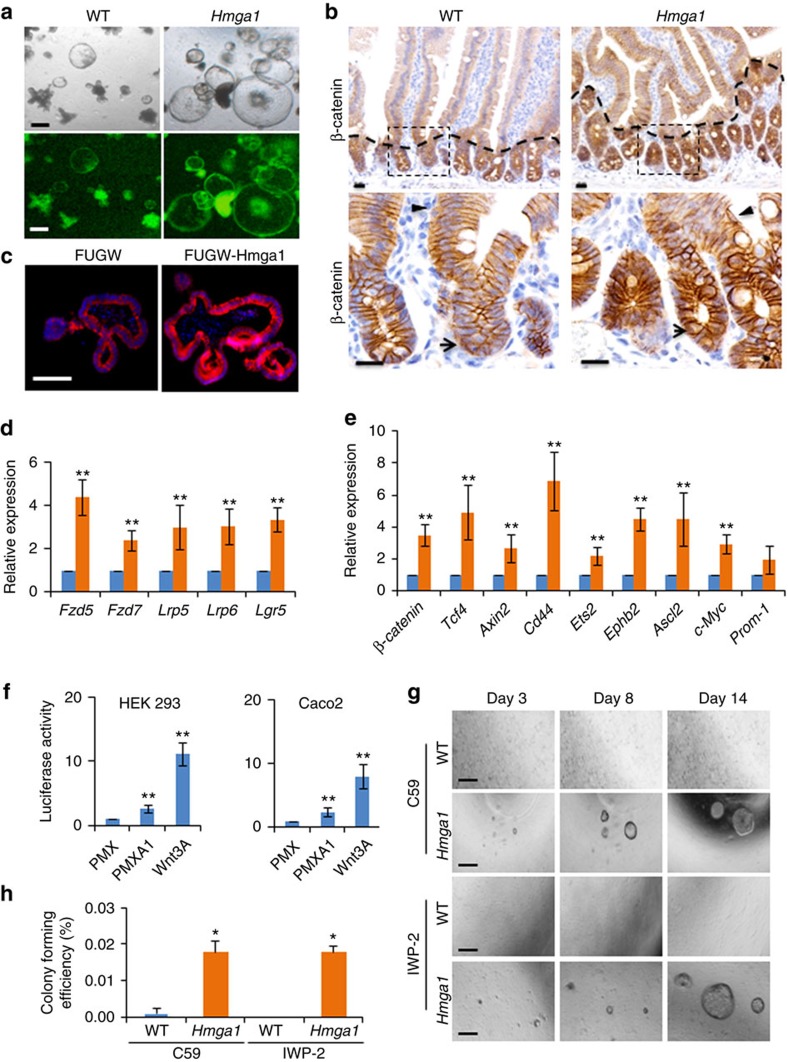
Hmga1 amplifies Wnt signalling in ISCs. (**a**) Representative organoids after exposure to Wnt3a (10 ng μl^−1^) are shown. *Hmga1* organoids form large, cyst-like spheres (standard (top) and fluorescent (bottom) microscopy). Scale bars, 50 μm. (**b**) β-Catenin IHC staining is shown in small intestines from WT and *Hmga1* mice. Scale bars, 20 μm. (**c**) β-Catenin IHC staining is shown in organoids engineered to express control lentivirus (FUGW; left) or *Hmga1* (FUGW-Hmga1; right). Scale bar, 20 μm. (**d**) Relative expression (mean±s.d.) of genes encoding Wnt agonist receptors was ascertained by qPCR in GFP+ ISCs from WT (blue) or *Hmga1* transgenic mice (orange) in two experiments performed in triplicate. *Gapdh* was used to control for loading. **P<0.01; two-tailed Student's *t*-test. (**e**) Relative expression (mean±s.d.) of genes encoding Wnt/Tcf4/β-catenin transcriptional targets was ascertained by qPCR in GFP+ ISCs from WT or *Hmga1* transgenic mice in two experiments performed in triplicate. *Gapdh* was used to control for loading. **P<0.01; two-tailed Student's *t*-test. (**f**) HEK 293 (left) and Caco2 (right) cells infected with the synthetic Wnt reporter construct containing seven optimal Tcf4/β-catenin-binding sites showed activation in cells overexpressing *Hmga1.* Purified Wnt3A (100 ng ml^−1^) protein was used as a positive control. Bars show mean luciferase activity±s.d. from two experiments performed in triplicate. **P<0.01; two-tailed Student's *t*-test. (**g**) Representative image of GFP+ ISCs from WT or *Hmga1* mice are shown in 3D culture with Wnt porcupine inhibitors IWP-2 (0.5 μM) and C59 (0.5 μM). Scale bars, 50 μm. (**h**) Relative colony-forming efficiency (mean±s.d.) from three experiments is shown with GFP+ ISCs from WT or *Hmga1* mice in 3D culture with the Wnt porcupine inhibitors (IWP-2, C59). *P<0.05; two-tailed Student's *t*-test.

When organoids from *Hmga1* mice were incubated with Wnt3a to enhance lentiviral transduction, we observed a striking phenotype whereby the *Hmga1* organoids formed very large, cyst-like, spherical organoids comprised predominantly of Lgr5+ ISCs, in contrast to WT organoids, which generated smaller cysts at a decreased frequency (Fig. 4a; Supplementary Fig. 6). These findings suggested that the *Hmga1* organoids are more sensitive to Wnt signalling. Wnt/Tcf4/β-catenin signalling is an evolutionarily conserved pathway important for self-renewal in epithelial crypt ISCs and many other tissue-specific adult stem cells^38^. Moreover, hyperactive Wnt signalling leads to malignant transformation in intestinal epithelium^38^. To begin to define the role of Hmga1 in Wnt/Tcf4/β-catenin signalling, we assessed immunostaining for β-catenin as a surrogate for canonical Wnt signalling. β-catenin was markedly increased in the *Hmga1* transgenic intestinal epithelium and concentrated at the base of the crypts (Fig. 4b). A similar increase was also observed in WT organoids transduced to overexpress *Hmga1* (Fig. 4c). Since Hmga1 functions as an architectural transcription factor that alters gene expression, we hypothesized that Hmga1 could upregulate expression of factors that enhance Wnt signalling. To test this, we first compared expression of genes encoding Wnt agonist receptors that function in intestinal epithelium, including *Lgr5*, *Frizzled* (*Fzd*)*5*, *Fzd7*, *low-density lipoprotein receptor-related protein 5 (Lrp5)* and *Lrp6* in purified Lgr5+ ISCs isolated from WT or transgenic mice. We found an increase in expression of all Wnt agonist receptors tested (*Lgr5*, *Fzd5/7*, *Lrp5/6*) in the *Hmga1* ISCs by >2 to 4-fold compared to control ISCs (Fig. 4d). Together, these results indicate that Hmga1 amplifies Wnt signalling by upregulating genes encoding Wnt agonist receptors in ISCs, including the ISC marker, *Lgr5*, in our model.

Once β-catenin is released from an inhibitory complex following Wnt signalling, it binds to DNA together with its partner, Tcf4, to induce Wnt pathway genes. In human embryonic stem cells, HMGA1 induces expression of *c-MYC* (ref. 16), a WNT/TCF4/β-catenin gene target, suggesting that Hmga1 could cooperate with Wnt/Tcf4/β-catenin to regulate the Wnt stem cell program. We therefore assessed expression of Wnt/Tcf4/β-catenin target genes, including *Axin2*, *Ascl2*, *β-catenin*, *Cd44*, *c-Myc*, *Ephb2*, *Ets*, *Tcf4* and *Prom-1*. Expression of all of these genes, excluding *Prom-1*, was increased by >2 to 6-fold in *Hmga1* ISCs versus WT control ISCs (Fig. 4e). Together, these results indicate that Hmga1 not only activates Wnt target genes together with Tcf4/β-catenin, but also amplifies Wnt signals by inducing expression of Wnt agonist receptor genes.

To better define the role of Hmga1 in regulating Wnt signalling, we cultured *Hmga1* organoids in the absence of the Wnt receptor agonist, R-spondin 1 (R-spo1), which is essential for organoid formation in this 3D culture system. R-spo1 is secreted by intestinal stromal cells and binds to the Lgr5 receptor to activate Wnt signalling^39^,^40^. We discovered a striking difference in the response of *Hmga1* organoids to the absence of R-spo1 as compared to the WT controls: *Hmga1* organoids continued to survive and proliferate for over 2 weeks, while the WT organoids lost their 3D structural organization and ultimately died by day 5 (Supplementary Fig. 7). By 3 weeks, however, the *Hmga1* organoids also stopped proliferating and lost their 3D organization, suggesting that *Hmga1* overexpression partially rescues loss of Wnt signalling via R-spo1, but does not completely recapitulate or bypass Wnt.

To further investigate the link between HMGA1 and Wnt, we determined whether cells overexpressing *HMGA1* have enhanced Wnt reporter activity using an established Wnt reporter construct that includes seven canonical Tcf4/β-catenin-binding sites in tandem upstream of a luciferase reporter gene^46^. Both HEK 293 human embryonal kidney cells and Caco-2 colon cancer cells were transduced with the Wnt reporter lentiviral vector, which includes a puromycin resistance gene. After selection for stable integration of the Wnt reporter construct by puromycin, the cultured cells were transduced with either control retrovirus or retrovirus expressing human *HMGA1*. In both cell types, HMGA1 activated Wnt reporter activity, while retroviral delivery of the control virus failed to do so (Fig. 4f). These experiments further support the role of HMGA1 in activating Wnt signalling.

To further test the link between Hmga1 and Wnt, we treated crypt cells with the Wnt inhibitor, C59, which blocks Wnt-mediated transcription and cell proliferation by inhibiting porcupine (PORCN), a protein required for Wnt palmitoylation, secretion and biological activity. Crypt cells isolated from *Hmga1* or WT small intestine were selected for small crypt size and cell number by passage through a 40 μM filter. The *Hmga1* crypts generated organoids and buds in C59 (0.25 μM) with a greater projected surface area than WT crypt cells cultured with C59 (Supplementary Fig. 8). In addition, the mean projected surface area of WT organoids cultured in C59 was reduced by days 6 and 10 compared to WT organoids cultured under control conditions with vehicle alone. In contrast, the *Hmga1* crypt cells were relatively impervious to C59; by day 6, there was no difference in projected surface area of *Hmga1* organoids compared to those cultured with vehicle alone, although the projected surface area decreased by day 10 when cultured with C59 compared to those cultured with vehicle control. This blunted response of *Hmga1* organoids to Wnt inhibition could result from the ability of Hmga1 to amplify Wnt signalling, thereby mitigating the effects of Wnt inhibition. Alternatively, or in addition to amplifying Wnt signalling, the *Hmga1* organoids could dampen the effects of Wnt inhibition by providing a buffer as a result of an increased size and cell number. Finally, an increase in the number of Paneth cells, which secrete Wnt agonists, could mitigate the effects of Wnt inhibition. To distinguish between these possibilities, we isolated single, purified Lgr5+ ISCs from transgenic or WT mice and cultured them in the presence of two different Wnt PORCN inhibitors, C59 and IWP-2. Strikingly, the *Hmga1* ISCs proliferated and formed 3D, cyst-shaped organoids for up to 10 days, albeit at a slower rate between days 6–10 compared to purified *Hmga1* ISCs cultured without inhibitor (Fig. 4g,h). In contrast, the WT ISCs generated very few organoids when cultured with C59. Moreover, no organoids formed from WT ISCs cultured in IWP-2 (Fig. 4g,h). Together, these findings demonstrate that *Hmga1* ISCs have a blunted response to Wnt inhibitors, which is consistent with their ability to amplify Wnt signals.

### Hmga1 expands the Paneth cell niche and upregulates *Sox9*

**Figure 5:**
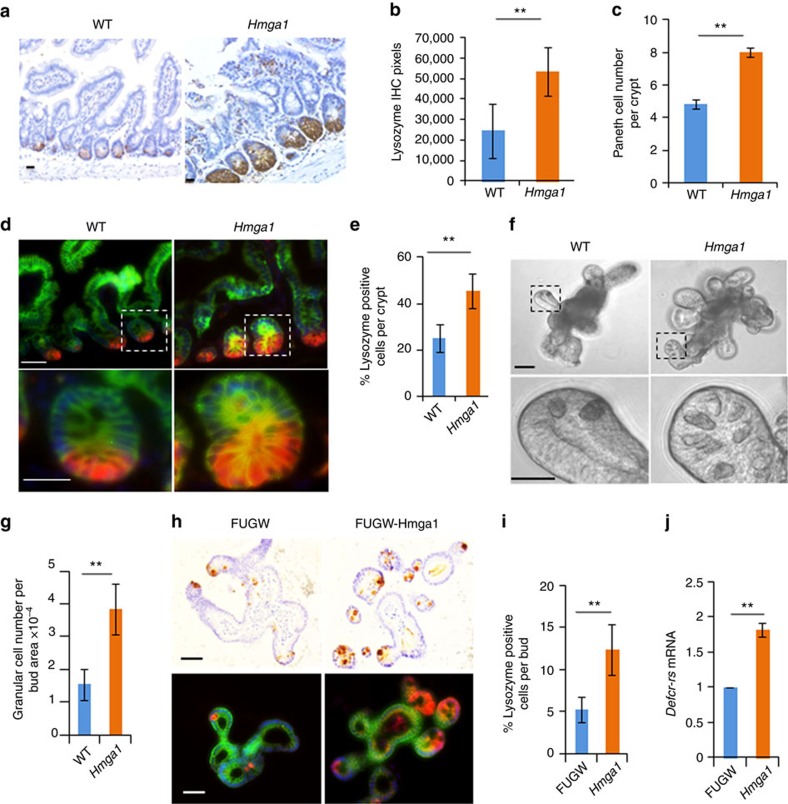
Hmga1 expands the Paneth cell niche. (**a**) IHC for lysozyme is shown from small intestines of WT and *Hmga1* transgenic mice. Scale bar, 20 μm. (**b**) Lysozyme IHC pixels (mean±s.d.; imagine pro-plus 6.0 software) is shown in small intestinal cross-sections (*n*=78 crypts per group; 3 mice per genotype). **P<0.01; Mann–Whitney test. (**c**) Paneth cell number (mean±s.d.) per crypt (*n*=100 crypts per group; 3 mice per genotype). **P<0.00001; Mann-Whitney test. (**d**) Immunofluorescent co-staining for lysozyme, Ep-CAM and DAPI is shown from small intestines of WT and *Hmga1* transgenic mice. Scale bars, 20 μm. (**e**) Paneth cell frequency (mean±s.d.) was obtained by dividing the Paneth cell number by total cell number per crypt in WT and *Hmga1* intestine (*n*=50 crypts per group; 3 mice per genotype). **P<0.00001; Mann–Whitney test. (**f**) Granular cells in WT and *Hmga1* organoid buds are shown via phase contrast microscopy. Scale bars, 50 μm. (**g**) Granular cells per bud PSA (mean±s.d.) from WT and *Hmga1* organoids are shown (*n*=35 buds per group). **P<0.00001; Mann–Whitney test. (**h**) IHC for lysozyme (top) and immunofluorescent co-staining for lysozyme, Ep-CAM and DAPI (bottom) are shown in organoids from WT or *Hmga1* mice. Scale bars, 50 μm. (**i**) Paneth cell frequency per bud (mean±s.d.) was calculated by dividing the Paneth cell number by the total bud cell number (*n*=30 organoids per group). **P<0.00001; Mann–Whitney test. (**j**) Relative expression (mean±s.d.) of *Defcr-rs,* the Paneth cell-specific transcript, is shown from two experiments performed in triplicate from organoids from WT and *Hmga1* mice. *Gapdh* was used to control for loading. **P<0.01; two-tailed Student's *t*-test.

Paneth cells are terminally differentiated epithelial cells derived from ISCs and located at the base of intestinal crypts^47^,^48^,^49^,^50^. They support ISC survival by secreting Wnt3a and other factors, thus providing an epithelial niche for ISCs. To determine whether Hmga1 alters the Paneth cell niche in our transgenic models, we stained for lysozyme using alkaline phosphatase, which marks Paneth cells. There was a marked increase in lysozyme stain (Fig. 5a), which was confirmed quantitatively by immunohistochemistry (IHC) pixels (Fig. 5b). To determine whether the increase in lysozyme stain corresponds to an increase in Paneth cell number, Paneth cells were enumerated in mouse intestinal tissue after controlling for the region of the intestine (Fig. 5c; *n*=100 crypts per group from duodenal epithelium). There was a marked increase in Paneth cell number per crypt, which paralleled the increase in lysozyme stain by IHC. Next, we co-stained the duodenal epithelium with EpCAM to demarcate cell borders, DAPI to indicate individual nuclei, and lysozyme such that the frequency of cells staining positive for lysozyme could be enumerated within the crypts (Fig. 5d). There was also a marked increase in Paneth cell frequency (Fig. 5e; *n*=50 crypts per group from duodenal epithelium), consistent with an expansion in the Paneth cell niche in the *Hmga1* transgenic duodenal epithelium as compared to WT control duodenal epithelium. To further confirm the increase in Paneth cells by Hmga1, we assessed Paneth cell number in *Hmga1* organoids using two different approaches. First, Paneth cells have a distinctive granular appearance on phase contrast microscopy. We therefore compared the mean number of granular cells per bud in WT and *Hmga1* organoids after controlling for bud size by dividing the Paneth cell number by the projected surface area per bud (Fig. 5f,g; *n*=35 buds per group). Similar to the *Hmga1* mouse intestinal tissue, there was a significant (*P*<0.00001; Mann–Whitney test) increase in Paneth cell number per bud projected surface area in the *Hmga1* organoids (Fig. 5g). Second, we assessed Paneth cell number by staining with lysozyme, which appeared to be increased in organoids transduced to express *Hmga1* compared to control organoids (Fig. 5h). To quantitatively assess the frequency of Paneth cells per total bud cell number, we co-stained with EpCAM, DAPI and lysozyme. There was an increase in Paneth cell frequency using this approach, similar to our results with intestinal tissue and granular cell number (Fig. 5h,i). Finally, to further corroborate this result, we assessed expression of a transcript that is highly specific for Paneth cells, *Defcr-rs*^51^, and found that it is increased in the *Hmga1* organoids compared to control organoids (Fig. 5j). Together, these results indicate that Hmga1 induces Paneth cell niche formation and expansion, which could help to promote ISC maintenance and expansion.

**Figure 6:**
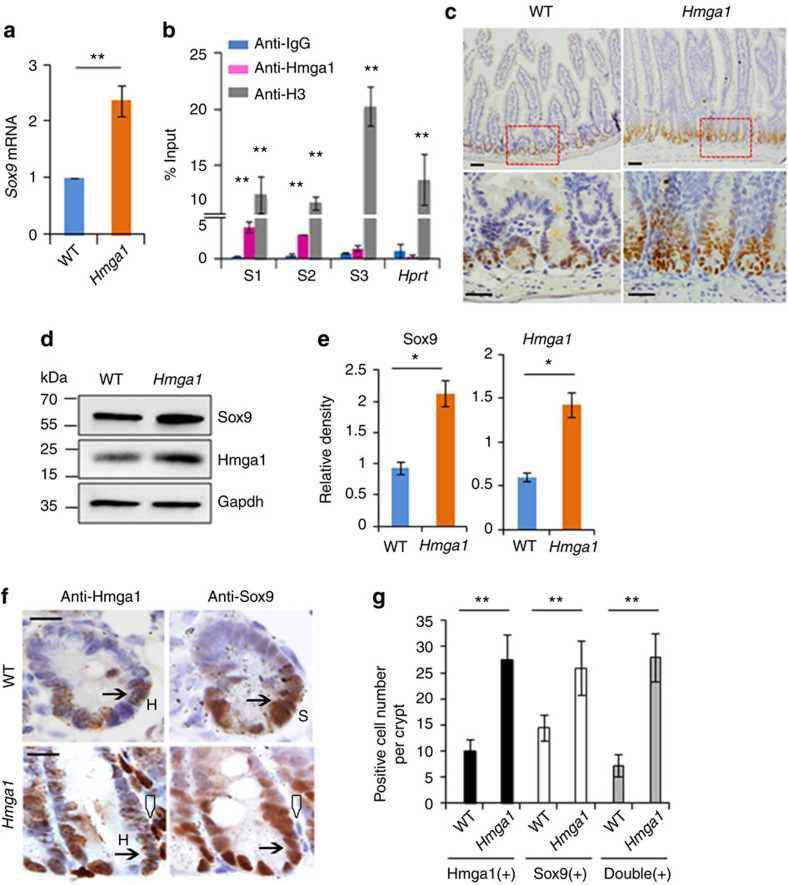
Hmga1 directly induces *Sox9* expression. (**a**) Relative *Sox9* mRNA (mean±s.d.) by qPCR in GFP^+^ ISCs from WT and *Hmga1* mice is shown from two experiments performed in triplicate. *Gapdh* was used to control for loading. **P<0.01; Student's *t*-test. (**b**) Hmga1 binding to the *Sox9* promoter by ChIP is shown from mouse crypt cells. IgG antibody and the *Hprt* promoter region were used as negative controls; histone H3 was a positive control. Bars show mean enrichment±s.d. from two experiments performed in triplicate; **P<0.01; two-tailed Student's *t*-test. (**c**) Sox9 IHC staining (brown) is shown in intestinal cross-sections from WT and *Hmga1* transgenic mice. Scale bars, 20 μm. (**d**) Western blots for Sox9, Hmga1, and Gapdh (loading control) were performed from freshly isolated crypt cells from WT and *Hmga1* transgenic mice. Western blots were done three times; a representative blot is shown. Size markers (kDa) are indicated. (**e**) Densitometry analysis±s.d. was performed on repeat western blots. *P<0.05; two-tailed Student's *t*-test. (**f**) Sox9 and Hmga1 IHC staining (brown) is shown in adjacent sections of small intestine from WT and *Hmga1* mice. Arrows show representative staining for Hmga1 (H) or Sox9 (S); triangles show *Hmga1* transgenic lymphocytes which have high levels of Hmga1 (but not Sox9). Scale bars, 50 μm. (**g**) Mean number of cells±s.d. staining positive for Hmga1, Sox9 or both were identified by IHC in adjacent cross-section on slides of small intestinal crypts from WT and *Hmga1* mice (*n*≥21 crypts per group.) **P<0.01 by Mann–Whitney test.

Because terminal differentiation to a Paneth cell requires Sox9 (refs 47, 48, 49), we hypothesized that Hmga1 fosters Paneth cell expansion by upregulating *Sox9* expression. HMGA1 induces the *SOX* family member, *SOX2*, in human embryonic stem cells^16^; further, both human and mouse *SOX9/Sox9* have similar AT-rich regions and predicted Hmga1 DNA-binding sites in the 5′ untranslated region. *Sox9* is also a β-catenin/Tcf4 target gene. We found that Hmga1 upregulates *Sox9* expression in the Lgr5+ ISCs as compared to WT Lgr5+ ISCs (Fig. 6a). To determine whether Hmga1 directly induces *Sox9* gene expression, we performed chromatin immunoprecipitation (ChIP) in intestinal crypt cells from WT mice. We discovered that Hmga1 binds directly to the *Sox9* promoter at two conserved sites (Fig. 6b; Supplementary Fig. 9a,b). Enrichment for Hmga1 binding was greatest at the proximal site (denoted site 1) and significant, albeit lower, at the second most proximal site (site 2; *P*<0.01 by two-tailed Student's *t*-test). Hmga1 binding was not enriched at the distal site (site 3). For a positive control, we used a histone H3 antibody, which showed significant enrichment (*P*<0.01 by two-tailed Student's *t*-test) at all promoters tested. For a negative control, we used the IgG antibody, which had minimal binding to chromatin at all promoter regions tested. As a second negative control, we used the murine *Hprt* gene; this promoter region was previously shown to be negative for Hmga1 occupancy. We confirmed that Hmga1 does not occupy the *Hprt* promoter (Fig. 6b). Next, we performed transfection experiments to determine whether Hmga1 transactivates the *Sox9* promoter reporter construct containing the Hmga1-binding site 1, which had the greatest enrichment for Hmga1 binding (Supplementary Fig. 9a–c). As a control, we included a *Sox9* promoter reporter construct lacking this Hmga1-binding site (Supplementary Fig. 9b,c). We found that Hmga1 induced the *Sox9* promoter construct that included site 1, while there was no induction of the promoter construct lacking this site (Supplementary Fig. 9d). As an additional control, we included a dominant-negative HMGA1 construct with mutations in the second AT-hook DNA-binding domain, which no longer binds to DNA^16^. The dominant-negative mutant also failed to transactivate either *Sox9* promoter construct (Supplementary Fig. 9d). To further validate this relationship between *Hmga1* and *Sox9*, we assessed *Sox9* mRNA in organoid cells transduced to overexpress *Hmga1* and found that *Sox9* was induced by *Hmga1* (Supplementary Fig. 9e). In contrast, we also found that *Sox9* was repressed in organoids with *Hmga1* knockdown following transduction with shRNA targeting *Hmga1* compared to controls (Supplementary Fig. 9f).

To determine whether cells with high levels of Hmga1 protein also have high levels of Sox9 protein, we assessed Sox9 protein and found that it is increased in the crypts of the *Hmga1* transgenic mice compared to WT controls by IHC (Fig. 6c). To quantitatively assess Hmga1 and Sox9 proteins, we performed western blot analysis of crypt cells from WT and transgenic mice; the *Hmga1* transgenic crypt cells had an increase in both Hmga1 and Sox9 protein compared to WT crypt cells (Fig. 6d,e). Next, we compared adjacent sections stained for Hmga1 or Sox9 and found that the majority of cells with detectable Hmga1 at the base of the crypts also have detectable Sox9 protein in both WT and transgenic mice (Fig. 6f,g). In WT mice, >70% of cells that stained positive for Hmga1 also stained positive for Sox9. In the transgenic mice, which have more cells with Hmga1 staining and higher levels of Hmga1, we found that >95% of cells that stained positive for Hmga1 also stained positive for Sox9 (Fig. 6f,g).

**Figure 7:**
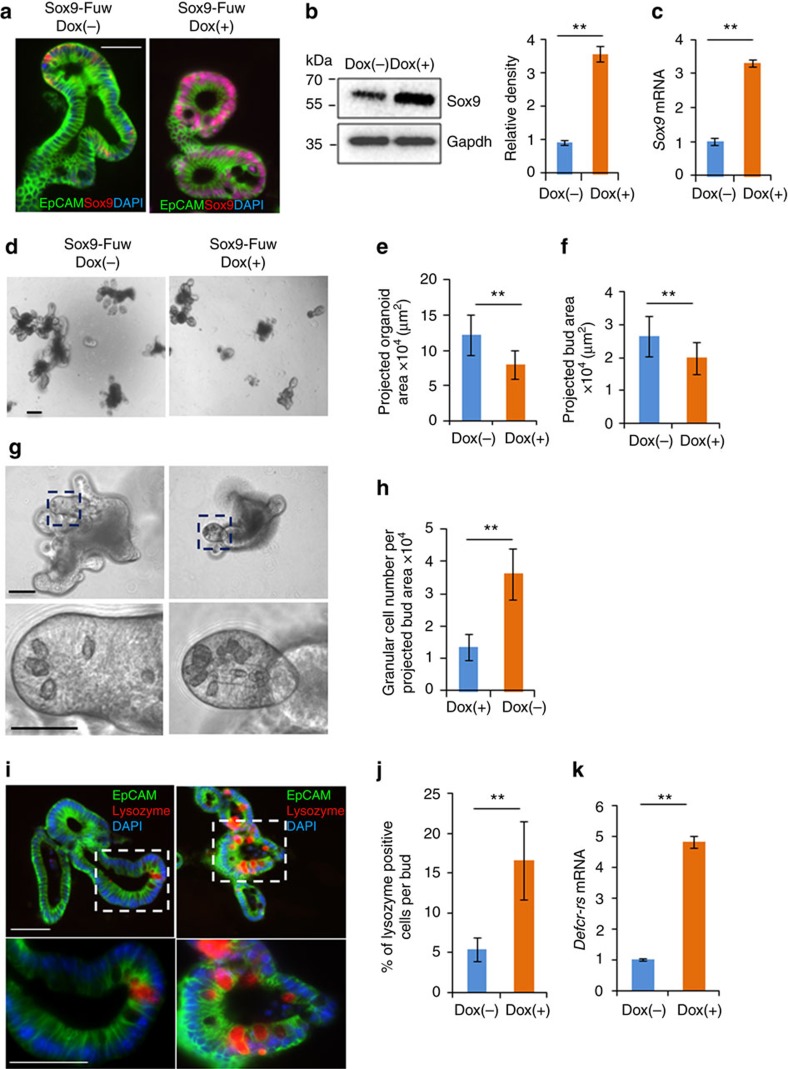
Paneth cell number increases in organoids overexpressing *Sox9*. (**a**) Immunofluorescent stain for Sox9 (red), DAPI (blue) and EpCAM (green) is shown in organoids transduced with lentivirus expressing doxycycline-dependent inducible *Sox9* (Sox9-Fuw). *Sox9* (Sox9-Fuw Dox(+)) was induced by doxycycline (1 μg ml^−1^) for 5 days; controls were cultured for 5 days without doxycycline. (**b**) Representative western blot for Sox9 in control and induced organoids is shown. Densitometry±s.d. was performed on three repeat western blots. **P<0.01; two-tailed *t*-test. (**c**) *Sox9* mRNA (mean±s.d.) was assessed by qPCR from two experiments performed in triplicate in control and induced organoids. *Gapdh* was used to control for loading. **P<0.01; two-tailed Student's *t*-test. (**d**) Typical organoids from uninduced controls (left) and induced (right) cultures are shown. (**e**) PSA±s.d. of organoids (*n*=52 per group) are shown. **P<0.01; two-tailed Student's *t*-test. (**f**) PSA±s.d. of buds (*n*=52 per group) are shown. **P<0.01; two-tailed Student's *t*-test. (**g**) Paneth cells identified as granular cells using phase contrast microscopy within individual buds are shown. (**h**) Granular cell frequency (mean±s.d.) was estimated by dividing the total granular cell number by the total bud PSA (*n*=52 per group). **P<0.01; Mann–Whitney test. (**i**) Paneth cells were identified based on lysozyme stain (red) in cells delineated by EpCAM (green) for cell membrane and (DAPI) for nuclei. (**j**) Paneth cell frequency (mean±s.d.) was ascertained by dividing the number of lysozyme staining cells (red) delineated by EpCAM (green) by the total cell number (DAPI) per bud (*n*=35 per group). **P<0.01; Mann–Whitney test. (**k**) Relative expression of *Defc*-*rs* (mean±s.d.) was compared in uninduced control and *Sox9-*induced organoids by qPCR from two experiments performed in triplicate. *Gapdh* was used to control for loading. **P<0.01; two-tailed Student's *t*-test. Scale bars, 50 μm.

While Sox9 is required for Paneth cell differentiation^47^,^48^,^49^,^50^,^51^, it is not known whether increased levels of Sox9 lead to expansion in Paneth cells. To test this, we transduced WT organoids with a lentivirus expressing *Sox9* following induction by doxycycline (Fig. 7). Immunofluorescent staining showed increased Sox9 protein in the doxycycline-induced organoids compared to uninduced control organoids cultured for the same period of time (Fig. 7a). Sox9 protein (by western blot analysis) and mRNA (by qPCR) were both increased relative to uninduced organoids (Fig. 7b,c). Consistent with the observation that Sox9 decreases proliferation *in vivo* in intestinal epithelium^49^, we observed that the organoid growth (estimated by projected surface area) was decreased in organoids with *Sox9* induction compared to uninduced organoids (Fig. 7d,e). We also found that the projected bud surface area was decreased with *Sox9* induction (Fig. 7f). Next, we compared Paneth cell frequency in *Sox9*-induced and uninduced organoids using two different approaches. First, we used phase contrast microscopy to identify Paneth cells morphologically by the distinctive granules, which are characteristic of these cells. The relative Paneth cell number per bud-projected surface area was increased in the *Sox9*-induced organoids compared to uninduced control organoids (Fig. 7g,h). Second, we used lysozyme stain to identify Paneth cells, together with EpCAM stain (to delineate cell membranes)^50^ and DAPI (to demarcate nuclei). There was also an increase in the frequency of Paneth cells per total bud cell number in *Sox9*-induced organoids (*n*>50 buds per condition from >50 organoids per condition; Fig. 7i,j). To further substantiate these results, we assessed expression of a transcript that is highly specific for Paneth cells, *Defcr*-*rs*^51^, and showed that it is also increased in the organoids overexpressing *Sox9* (Fig. 7k). These results suggest that Hmga1-mediated induction of *Sox9* helps to expand the Paneth cell niche, although there are likely to be other factors that contribute to Paneth cell differentiation. Together, our results demonstrate that Hmga1 drives self-renewal and expansion of ISCs in addition to helping to establish an epithelial stem cell niche through Paneth cell differentiation.

### *HMGA1* and *SOX9* in colonic epithelium and colorectal cancer

**Figure 8:**
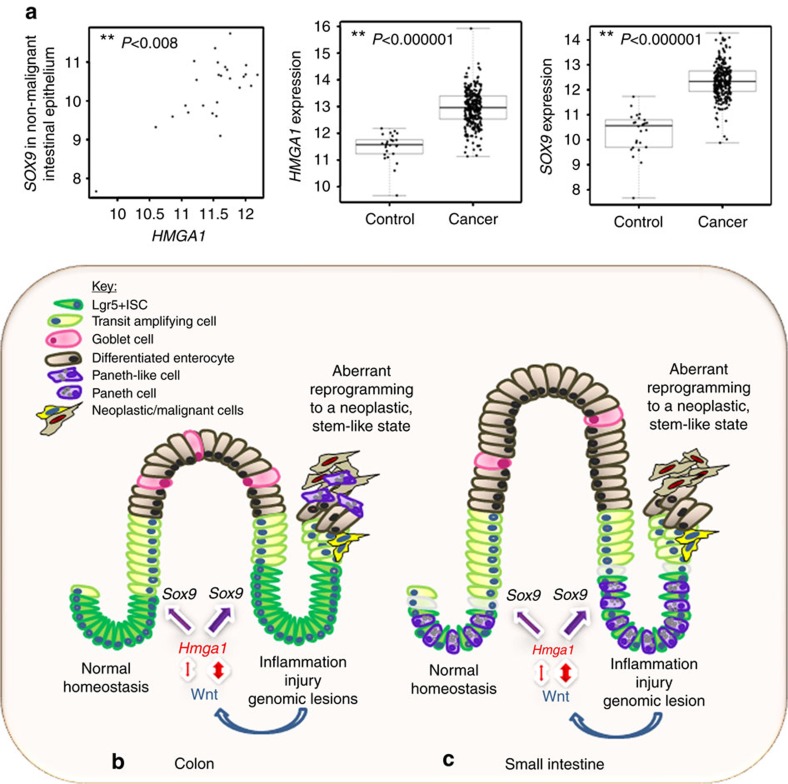
Hmga1 in normal intestinal homeostasis and reprogramming to neoplasia. (**a**) There is a significant positive correlation (*r*=0.51; *P*=0.008) between *HMGA1* and *SOX9* in control, non-malignant large intestinal epithelium by Spearman's correlation (*n*=26). There is also a highly significant upregulation of both *HMGA1* and *SOX9* in colorectal cancer (*n*=293, *P*<0.000001). Boxplots, scatterplots and Spearman rank-based correlations were calculated using R statistical software. (**b**) Model depicting normal ISC function and tissue homeostasis in the large intestine under conditions of tightly regulated *Hmga1* expression and Wnt signalling (left). Intestinal epithelium homeostasis is disrupted (right) in the setting of aberrant *Hmga1* expression, leading to expansion in the ISC compartment, excessive Wnt signalling, and abnormal proliferation, culminating in epithelial reprogramming to neoplastic, transformed cells. (**c**) Model depicting normal ISC function and tissue homeostasis in the small intestine under conditions of tightly regulated *Hmga1* expression, Paneth cell differentiation and Wnt signalling (left). Expansion in the ISCs and Paneth cell niche occurs when homeostasis is disrupted (right) in the setting of aberrant *Hmga1* expression. This contributes to amplified Wnt signalling, abnormal proliferation, epithelial reprogramming and neoplastic transformation.

To determine whether our findings in mouse intestine are relevant to humans, we assessed expression of *HMGA1* and *SOX9* in human intestinal epithelium. Using the Cancer Genome Atlas (TCGA), we found that *HMGA1* and *SOX9* are positively correlated in normal colonic epithelium (*P=*0.008, *r*=0.52; Fig. 8a). We also stained human small intestine, and found that HMGA1 localizes to the columnar basal cells within the crypts where ISCs are located (Supplementary Fig. 10). Because *HMGA1* is overexpressed in diverse epithelial cancers and correlates with cancer stem cell properties in experimental models, we also sought to determine whether *HMGA1* and *SOX9* are co-regulated in colorectal cancer. Strikingly, both *HMGA1* and *SOX9* are markedly upregulated in human colorectal cancer (*P<*0.0000001), although their expression was not correlated in this setting (Fig. 8a). Interestingly, the relative expression and amplitude of *HMGA1* mRNA levels are greater than that of *SOX9* (Fig. 8a). These data support a model whereby *HMGA1* and *SOX9* are crucial for normal ISC function, and both become upregulated in carcinogenesis (Fig. 8b,c). These findings also suggest that upregulation of both *HMGA1* and *SOX9* beyond a threshold may be necessary for early reprogramming of an epithelial cell to a neoplastic cell, while further increases in *HMGA1* could drive tumour progression.

## Discussion

*HMGA1* expression has been identified among genes most enriched in embryonic and adult stem cells, although its function in these settings had been poorly understood. Our studies reveal a role for Hmga1 in both stem cell self-renewal and establishment of a stem cell niche within small intestinal crypts. The *HMGA* gene family includes *HMGA1* (on chromosome 6p21) and *HMGA2* (on chromosome 12q15), both of which are highly expressed during embryonic development, but with low or undetectable levels in differentiated tissues^13^,^16^,^25^,^26^. Aberrant expression of *HMGA1* occurs in most poorly differentiated human cancers, including gastrointestinal cancers such as colon, gastric, pancreatic and esophageal cancers, and high levels correlate with poor outcomes in diverse tumours^13^,^30^,^31^,^32^,^33^,^34^,^35^. Notably, a prior study in colorectal cancer identified *HMGA1* among the genes most enriched in cancer relative to adjacent, non-malignant tissue^33^. *HMGA1* is also required for properties attributed to cancer stem cells, including tumour initiator cells, growth as 3D spheres and metastatic progression^14^,^18^. In contrast, *HMGA2* overexpression occurs primarily in benign tumours of mesenchymal origin^31^, as well as a subset of malignant tumours^52^,^53^,^54^,^55^. The dual role for Hmga1 in normal development and poorly differentiated cancers suggests that it regulates cell fate decisions, although a detailed understanding of molecular mechanisms involved in these processes was previously unknown.

Here we show that *Hmga1* amplifies Wnt signalling to drive self-renewal and ISC expansion. Hmga1 not only upregulates Wnt agonist receptor genes, but also enhances expression of genes downstream of WntTcf4/β-catenin. Our results, together with the prior finding that Tcf4 binds to the *HMGA1* promoter in colorectal cancer cells^56^, suggest that Hmga1 is involved in a ‘feed-forward' loop, whereby Tcf4/β-catenin induces *Hmga1*, leading to enhanced Wnt signalling. The expansion of ISCs in our transgenic mouse model was recapitulated *in vitro* in organoid cultures, which depend on Hmga1 for organization into 3D structures and bud formation. Our transgenic mouse and organoid models provide valuable tools to further dissect downstream pathways regulated by Hmga1 in ISCs. In a murine model of gastric cancer, Wnt signalling also upregulates *Hmga1* (ref. 57). We are the first to show that Hmga1 enhances Wnt/β-catenin signalling at multiple levels in the pathway, consistent with a feed-forward amplification loop, whereby Wnt induces *Hmga1*, which in turn, upregulates Wnt/β-catenin signalling (Fig. 7).

Our work also uncovered a role for Hmga1 in Paneth cell differentiation, mediated in part, through *Sox9.* Paneth cells constitute an epithelial niche, providing Wnt3a and other signals to maintain ISCs and permit self-renewal within the crypts. Cues from the epithelial and stromal niche are likely to help govern whether Hmga1 induces *Sox9* to drive Paneth cell differentiation or other Wnt signals to drive self-renewal. Because adult stem cells can divide asymmetrically, Hmga1 could promote ISC division to generate both an identical daughter cell and a differentiated Paneth cell, or even a more fully differentiated transit-amplifying cell. Recent studies indicate that R-spondin 1, another Wnt receptor agonist secreted by intestinal stromal cells, is required for ISC maintenance in mice^39^,^40^. It remains to be seen whether Hmga1 also regulates R-spondin 1 in stromal niche cells. Of note, mesenchymal stem cells within the bone marrow niche also express high levels of *HMGA1*, although the functional significance of HMGA1 in this setting is not yet known^58^. Prior studies indicate that quiescent, label-retaining cells that are responsible for intestinal epithelial regeneration following injury also differentiate into secretory progenitors and Paneth cells^59^. Hmga1 may also function in this setting, although lineage-tracing experiments will be needed to test this.

In addition to establishing a role for Hmga1 in maintaining ISC and niche compartments, we also found a positive correlation between *HMGA1* and *SOX9* in normal human large intestinal epithelium. Moreover, both genes are upregulated in colorectal cancer, indicating that this pathway may contribute to human intestinal carcinogenesis^14^,^33^. A recent study in the *Adenomatous Polyposis Coli* (*Apc*)^+/−^ murine model of intestinal carcinogenesis showed that *Hmga1* is downstream of the miR-26 tumour suppressor^60^, suggesting that *APC* mutations in colorectal cancer lead to *HMGA1* induction through loss of miR-26. *HMGA1* is also upregulated in the setting of inflammation^13^, and intestinal carcinogenesis is frequently preceded by chronic inflammation and injury^61^. Thus, both inflammatory lesions and genetic alterations (*APC* inactivating mutations) could cooperate to induce *HMGA1* during carcinogenesis. Paneth cell expansion also occurs in mice with deletion in the *Apc* tumour suppressor^62^, and Hmga1 could contribute to this phenotype. Although there are no discrete populations of Paneth cells outside of the proximal colon in humans, lysozyme-expressing Paneth-like cells are found in human adenomas^62^. Based on our findings, it is plausible that HMGA1 induces formation of Paneth-like niche cells during colon carcinogenesis in the intestinal epithelium. Studies in murine pancreatic cancer show that *SOX9* reprograms acinar cells to ductal cells during carcinogenesis^63^. Similarly, HMGA1 could induce *SOX9* and enforce Wnt signalling to drive stem cell properties and reprogram intestinal epithelial cells during carcinogenesis. Although *HMGA1* and *SOX9* are positively correlated in normal colonic epithelium and both become upregulated in cancer, it is not surprising that the correlation is lost in colorectal cancer. This is consistent with the large body of evidence demonstrating that colon cancer is initiated by the progressive acquisition of mutations in the APC pathway, which include genes upstream of *SOX9* and the *SOX9* gene itself^10^,^11^. Thus, there are multiple distinct mechanisms that could give rise to overexpression of *HMGA1* and *SOX9* in colorectal cancer, and *SOX9* may depend, at least in part, on genetic lesions distinct from *HMGA1* overexpression in this setting. Together, our work not only reveals a novel function for Hmga1 in intestinal homeostasis through self-renewal of ISCs and Paneth cell differentiation, but also sheds light on possible mechanisms involved in Hmga1-mediated neoplastic transformation and intestinal carcinogenesis.

## Methods

### Mouse models

The *Hmga1* transgenic construct and mice have been previously described^35^,^36^. Female Lgr5-eGFP-IRES-CreERT2 mice^6^ (Jackson Labs) were crossed with male *Hmga1a* transgenics^35^. All mice were on the C57Bl6 background. Animal experiments were conducted in accordance with our institutional Animal Care and Use Committee (protocol# MO14M187). All mice were housed in a sterile environment where they had free access to food and water as outlined in our institutional guidelines. The ages of the mice were 2–4 months; ∼50% were male. Experiments comparing wild-type to transgenic mice were matched for age and gender in all cases.

### Crypt isolation

Crypts were isolated using an established protocol^4^,^5^,^6^,^7^,^41^. First, intestines were flushed with phosphate-buffered saline (PBS) and incised longitudinally after which villi were removed mechanically by scraping. Sections (1 cm) were incubated in EDTA (5 mM)/PBS for 15 min at 4 °C per fraction of epithelium. After incubation, the epithelium was separated by vigorous shaking and the remaining intestinal tissue was placed in a new tube for collection of subsequent fractions. After isolation, crypt cells were pelleted, passed through a 70 μm cell strainer (unless indicated otherwise), evaluated for purity microscopically and counted. For purification of single Lgr5-GFP+ ISCs, isolated crypts were incubated in culture medium for 45 min at 37 °C, followed by trituration with a glass pipette. Dissociated cells were passed through a cell strainer with a pore size of 20 μm. GFP+ and GFP− cells were sorted by FACS Aria model (BD Biosciences).

### Organoid culture and re-plating assay

Mouse organoids were established and maintained from isolated crypts of the proximal small intestine or from Lgr5+ISCs isolated as single cells^4^,^5^,^6^,^7^,^8^,^41^. The basic culture medium (advanced Dulbecco's modified Eagle's medium/F12 supplemented with penicillin/streptomycin, 10 mmol l^−1^ HEPES, 13 Glutamax, 13 B27 (all from Life Technologies), and 1 mmol l^−1^ *N*-acetylcysteine (Sigma)) was supplemented with 50 ng ml^−1^ murine recombinant EGF (Peprotech), R-spondin 1 (1 μg ml^−1^) and Noggin (10 ng ml^−1^). Wnt inhibitors C59 and IWP-2 are commercially available (Abcam). Conditioned media was produced using HEK 293T cells stably transfected with HA-mouse Rspo1-Fc (a gift from Calvin Kuo, Stanford University). Advanced DMEM/F12 media supplemented with penicillin/streptomycin, 10 mmol l^−1^ HEPES, and 13 Glutamax was conditioned for 1 week. For the re-plating assay, an equal number of cells from each model were isolated by mechanical dispersion with a 10 ml pipet after 3–5 days in culture. Cells were then counted and re-plated using the conditions described above.

### Lentivirus and transduction

The FUGW control vector (Addgene plasmid # 14883) and FUGW-Hmga1 lentiviral vectors^36^,^64^ have been described^14^,^16^,^18^. The shRNA interference plasmid for Hmga1 (#TRCN0000182169; the RNAi Consortium/TRC) has been described^14^,^16^,^18^. The empty shRNA vector was used as a control. For inducible silencing, pTRIPz-Hmga1-shRNA linked to RFP reporter was engineered to be inducible by tetracycline or analogues (Tet-On) and produces tightly regulated induction of shRNA expression in the presence of doxycycline (0.5 μg ml^−1^). The annealed Hmga1-shRNA oligonucleotides were cloned into linearized pTRIPZ empty vector (Open Biosystems catalogue number RHS4750). For pTRIPz-Hmga1-shRNA transductions, puromycin (2 μg ml^−1^) was added to media after 2–3 days for selection. For all lentiviral transductions, we modified an established protocol^65^ using magnetic nanoparticles (ViroMag R/L, OZ Bioscience, Inc.) and a magnetic plate (ViroMag R/L, OZ Bioscience, Inc., catalogue number: MF10000) to transduce crypt cells and organoids. Organoid fragments were seeded with 150 μl transduction medium into 48-well plates. Virus was added with viroMag R/L solution for 15 min at room temperature (2,500–3,000 virus particles per cells) to cells. The cell culture plate was placed on the magnetic plate for 30–60 min in a 37 °C tissue culture incubator. Cells were then incubated overnight at 37 °C. Organoid fragments and transduction media were then transferred to a 1.5 ml tube for centrifugation at 900 *g* for 5 min. The supernatant was discarded and tubes containing the pellet was placed on ice for 5 min. Next, 120 μl of matrigel was added and the pellet was resuspended by pipetting slowly up and down. Drops (30 μl) of basement matrix–cell mixtures were seeded into a new 48-well plate and incubated at 37 °C for 5–15 min until the basement matrix solidified. Media (ENRWntNic)^6^ was then added to each well and placed into a tissue culture incubator. Common ENR media^6^ was used and changed every 2–3 days beginning 4–6 days after the transduction.

### Gene expression analysis and chromatin immunoprecipitation

Wnt signalling molecule gene expression was detected by qPCR^14^,^15^,^16^ (see Supplementary Table 1 for primer sequence). Reactions were performed using the SYBR Green PCR Master Mix (Applied Biosystems) with the ABI 7500 qRT–PCR machine. *Gapdh* mRNA was assessed as the loading control^14^. For ChIP, primers were designed using the sequence data from MatInspector *in silico* transcription factor-binding site prediction algorithm^66^ with amplicon sizes of around 200 bp. Both positive control antibodies (histone H3) and negative control antibodies (IgG) as well as a negative control promoter sequence (from the *Hprt* gene) were included^36^,^67^. (See Supplementary Table 2 for ChIP primer sequences and antibodies.) All experiments were done in triplicate and performed at least twice.

### Western blots

Total protein from organoid cultures or tissues was isolated using RIPA buffer (Sigma, USA). Lysates were centrifuged and separated on a gradient gel (8–12%) via SDS–polyacrylamide gel electrophoresis, blotted onto a membrane (polyvinylidene fluoride; Bio Rad, USA), and analysed using specific antibodies (Supplementary Table 3). Bands were visualized using enhanced chemiluminescence (ECL Kit, Amersham Biosciences, USA). Images of all uncropped western blots can be found in Supplementary Fig. 11.

### Statistical analysis

Data from all experiments were assessed for normal distribution (Ryan–Joyner and D'Agostino–Pearson tests), and when normally distributed, compared using the two-tailed, Student's *t*-test. The Mann–Whitney test was used for data that were not normally distributed. *P<*0.05 was considered significant. Experiments that were technical failures, such as *in vitro* cultures in which controls did not grow, were excluded from the statistical analysis. Sample sizes were chosen empirically based on previous experiments; no statistical methods were used to predetermine sample sizes. Investigators were not blinded. Mice were analysed based on genotype; randomization was not performed.

### TCGA gene expression analysis

Transcript abundance for *HMGA1* and *SOX9* from human RNA-Seq data sets obtained from 293 primary colorectal cancers and 26 non-malignant colonic epithelial samples were assessed using RNA-Seq expectation maximization (RSEM) normalization. IluminaGa RNA-Seq data are available through TCGA data portal (Archive: COADREAD.rnaseqv2__illuminaga_rnaseqv2__unc_edu__Level_3__RSEM_genes_normalized__data.data.txt). Expression levels were converted to log (base 2) after adding a small quantity (0.001) to account for zeros in the data. Boxplots, scatterplots and Spearman rank-based correlations were calculated using R statistical software suite using standard functions^67^.

### Immunohistochemistry

Haematoxylin & eosin and IHC staining of organoid and intestinal sections were performed after fixing organoid cultures in formalin at 4 °C before paraffin or frozen embedding^14^,^16^,^68^,^69^. (See Supplementary Table 3 for list of primary antibodies.)

### Time-lapsed confocal imaging and image analysis

Crypt cells were isolated from WT and Hmga1 mice separately, resuspended in matrigel, and cultured on glass-bottom 24-well plates^41^. Crypt cells were fixed either immediately (day 0) after seeding or following culture for 6 days^42^,^43^,^44^,^45^. Green fluorescence of Lgr5+ (GFP+) cells was amplified by labelling anti-GFP antibody (00-106-215, Rockland Immunochemicals Inc. Limerick, PA) followed by staining with Alexa Fluor 488 anti-Goat secondary antibody (A-11055, ThermoFisher Scientific, Waltham, MA). Entire organoids were visualized with Alexa Fluor 568 Phalloidin (A12380, ThermoFisher Scientific) and Hoescht 33258 labelling (H3569, ThermoFisher Scientific) using confocal imaging^42^,^43^,^44^,^45^. Images were collected using a Solamere Technology Group spinning disk confocal microscope with a × 10 objective (Zeiss Microimaging). A combination of μManager and Piper (Stanford Photonics) imaging software was used for imaging acquisition^42^,^43^,^44^,^45^. All organoids were imaged using identical excitation and detection settings per fluorescence channel. ImageJ was used for post-acquisition image analysis and quantification of Lgr5+ (green channel) Z-projected surface areas (μm^2^) per organoid. Briefly, 2 μm-step Z-stacks of single organoids were contrasted to exclude background fluorescence, maxima-projected to create a single plane image per channel, after which a threshold was applied for specific signal within organoid boundaries. Projected monochromatic surface area was then measured within each imaged organoid, and expansion rate of Lgr5+ cell population in WT and *Hmga1* mice, separately, was calculated by normalization of day 6 projected area over day 0 projected area in the green channel.

### Cell culture

Caco-2 and HEK 293 cell lines (from ATCC) were cultured in high-glucose DMEM, supplemented with 4 mM glutamine, 50 U ml^−1^ penicillin, 50 μg ml^−1^ streptomycin (all from Invitrogen) and 10% fetal bovine serum. All cell lines were routinely tested for mycoplasma contamination with the MycoAlert Mycoplasma Detection Kit (Lonza). For cell line authentication, short-tandem repeat analysis was performed on isolated genomic DNA with the GenePrint 10 System from Promega, and peaks were analysed using GeneMarker HID from Softgenetics. Allele calls were searched against short-tandem repeat databases maintained by ATCC (www.atcc.org) and DSMZ (www.dsmz.de).

### Data availability

The TCGA RNA-seq data (Fig. 8a) have been deposited in EZID (at the California Digital Library) with identifier: Broad GDAC Firehose stddata__2013_08_09 run, doi:10.7908/C17W6BB3. Relevant primary data are presented in the manuscript and supplementary files; all primary data are available from the authors on request.

## Additional information

**How to cite this article:** Xian, L. *et al*. HMGA1 amplifies Wnt signalling and expands the intestinal stem cell compartment and Paneth cell niche. *Nat. Commun.* **8**, 15008 doi: 10.1038/ncomms15008 (2017).

**Publisher's note:** Springer Nature remains neutral with regard to jurisdictional claims in published maps and institutional affiliations.

## Supplementary Material

Supplementary Information Supplementary Figures, Supplementary Tables.
