# Supplementary material for: HMGA1 amplifies Wnt signalling and expands the intestinal stem cell compartment and Paneth cell niche
Source: Nat Commun. 2017 Apr 28;8:15008. doi: 10.1038/ncomms15008 (PMC5414379; doi:10.1038/ncomms15008)
Supplement: Supplementary Information — Supplementary Figures, Supplementary Tables. [file ncomms15008-s1.pdf]

Supplementary information

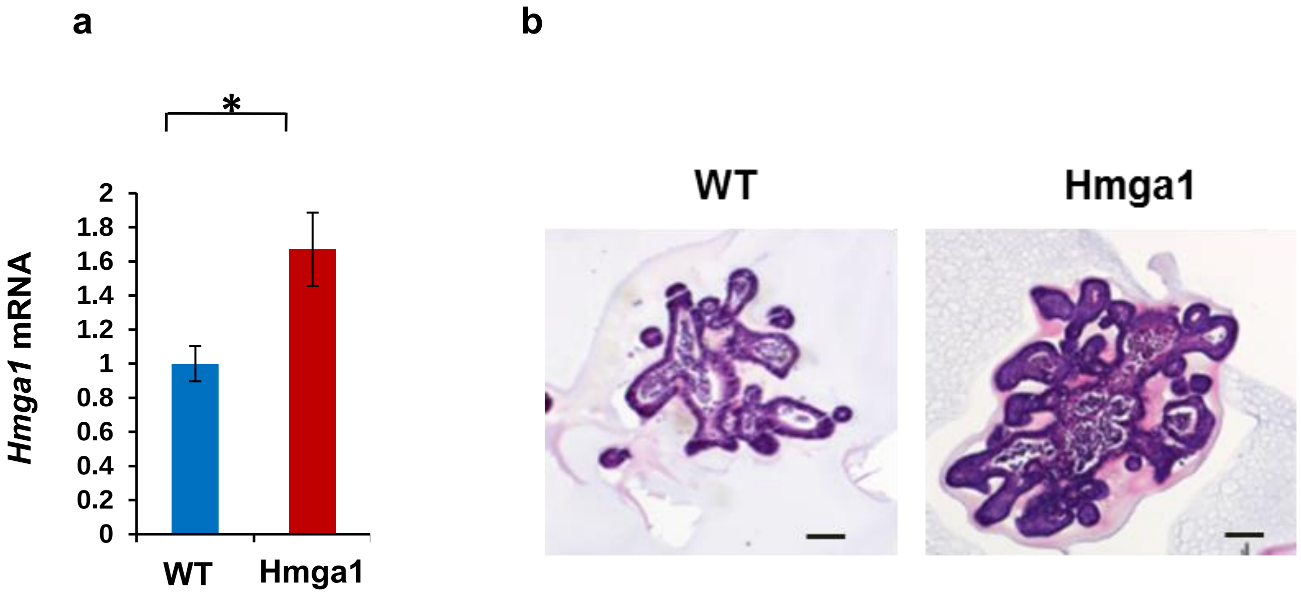

Supplementary Figure 1. *Hmga1* transgenic crypt cells overexpress *Hmga1* and generate larger 3D organoids with enhanced bud formation.

(a) Relative *Hmga1* expression was assessed by qPCR in the organoids derived from WT or *Hmga1* transgenic mice at 4 months. *Gapdh* expression was used to control for loading. **P*<0.05 (student's t-test; error bars represent standard deviations.)

(b) *Hmga1* organoids were larger and generated more buds, as shown here by haematoxylin & eosin (H & E). Scale bar: 50 µm

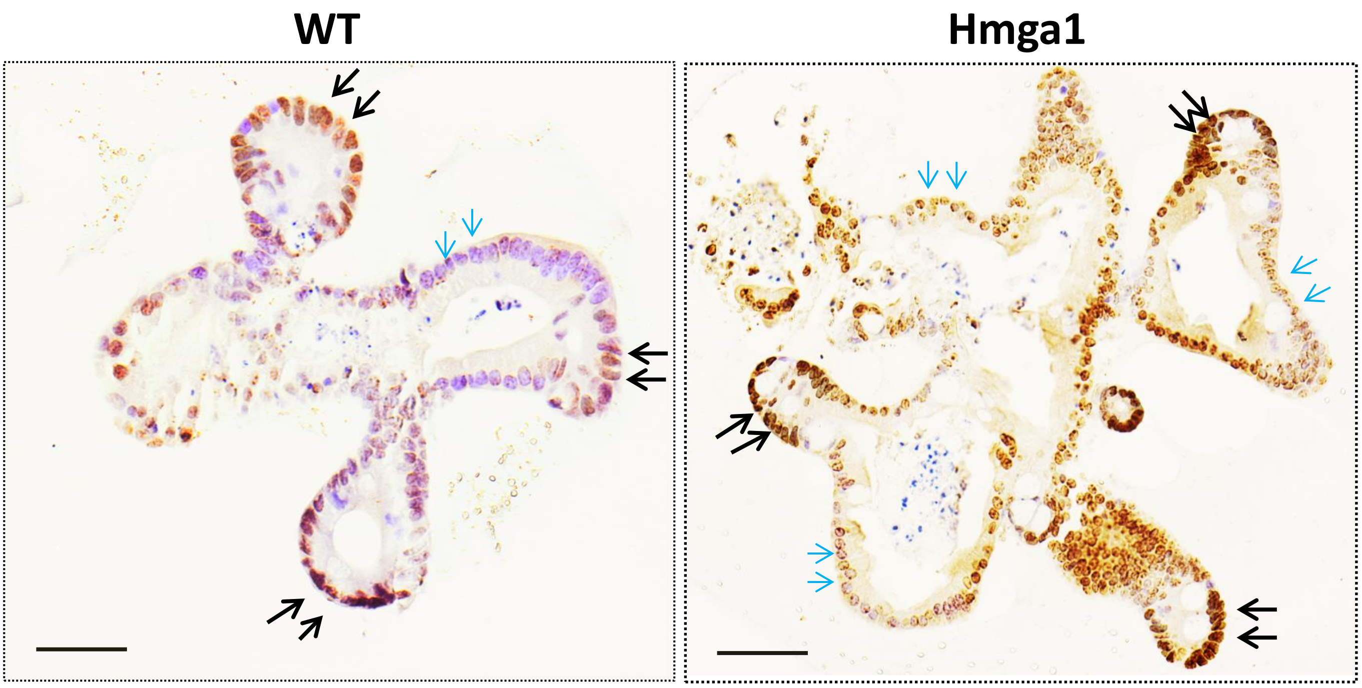

Supplementary Figure 2. Hmga1 immunohistochemical staining in organoids from pooled crypt cells from WT or transgenic mice. Immunohistochemical (IHC) staining was performed for Hmga1 (brown) in organoid sections derived from 4 month-old WT or *Hmga1* transgenic mice. Black arrows indicate intense staining at the bud tips; the blue arrows indicate decreased or absent staining in more differentiated cells. Scale bar: 50 µm

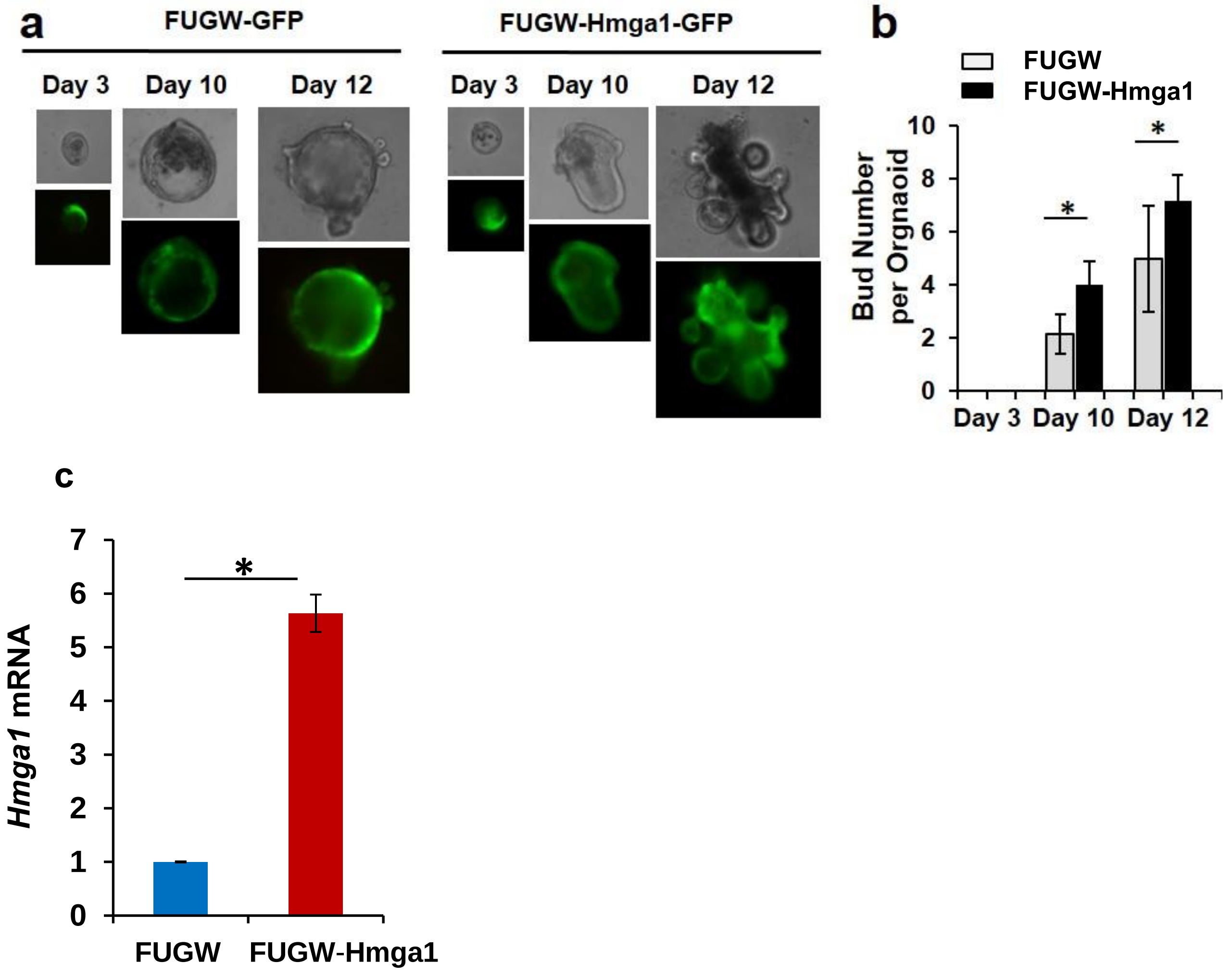

Supplementary Figure 3. *Hmga1* enhances bud formation in 3D organoid cultures.
(a) Representative images of WT organoids transduced with control lentivirus (FUGW; left) or lentivirus overexpressing *Hmga1* (FUGW-Hmga1; right) are shown.
(b) Mean bud number per organoids were determined at days 3, 10 and 12 following transduction with control lentivirus (FUGW) or lentivirus expressing *Hmga1* (FUGW-Hmga1). *P<0.05 (student's t-test, n=6/group; error bars represent standard deviations)
(c) Relative *Hmga1* expression in the WT organoids transduced with control lentivirus (FUGW) or lentivirus overexpressing *Hmga1* (FUGW-Hmga1) was assessed 21 days after transduction by qPCR. *Gapdh* expression was used to control for loading. *P<0.05 (student's t-test; error bars represent standard deviations)

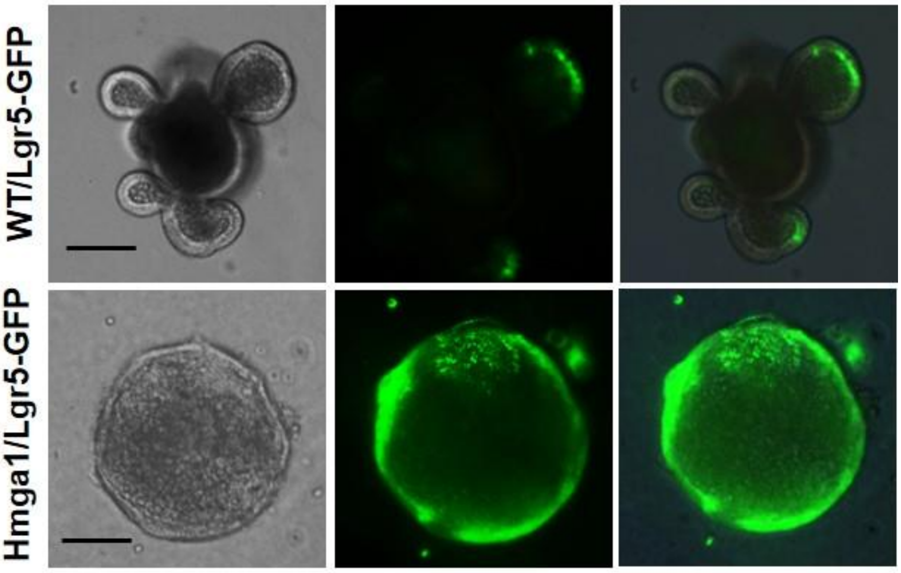

Supplementary Figure 4. Organoids derived from *Hmga1* Lgr5-GFP+ cells adopt a cyst-like morphology and remain undifferentiated in 3D culture. Organoids derived from *Hmga1* Lgr5-GFP+ ISCs adopt a cyst-like morphology similar to that of Wnt-treated organoids after 2 weeks in culture and maintain the Lgr5+ ISC marker.

Left panels: Phase contrast microscopy shows a typical organoid image from each model.

Middle panels: Fluorescent image (GFP) image

Right Panels: Merged image; the fluorescent images show that the majority of cells in the *Hmga1* organoids remain Lgr5-GFP+ ISCs, while organoids generated from WT Lgr5-GFP+ ISCs generate more typical organoids with buds containing GFP+ ISCs at the tips and more differentiated, GFP- cells at the base of the buds. Scale bar: 100 µm

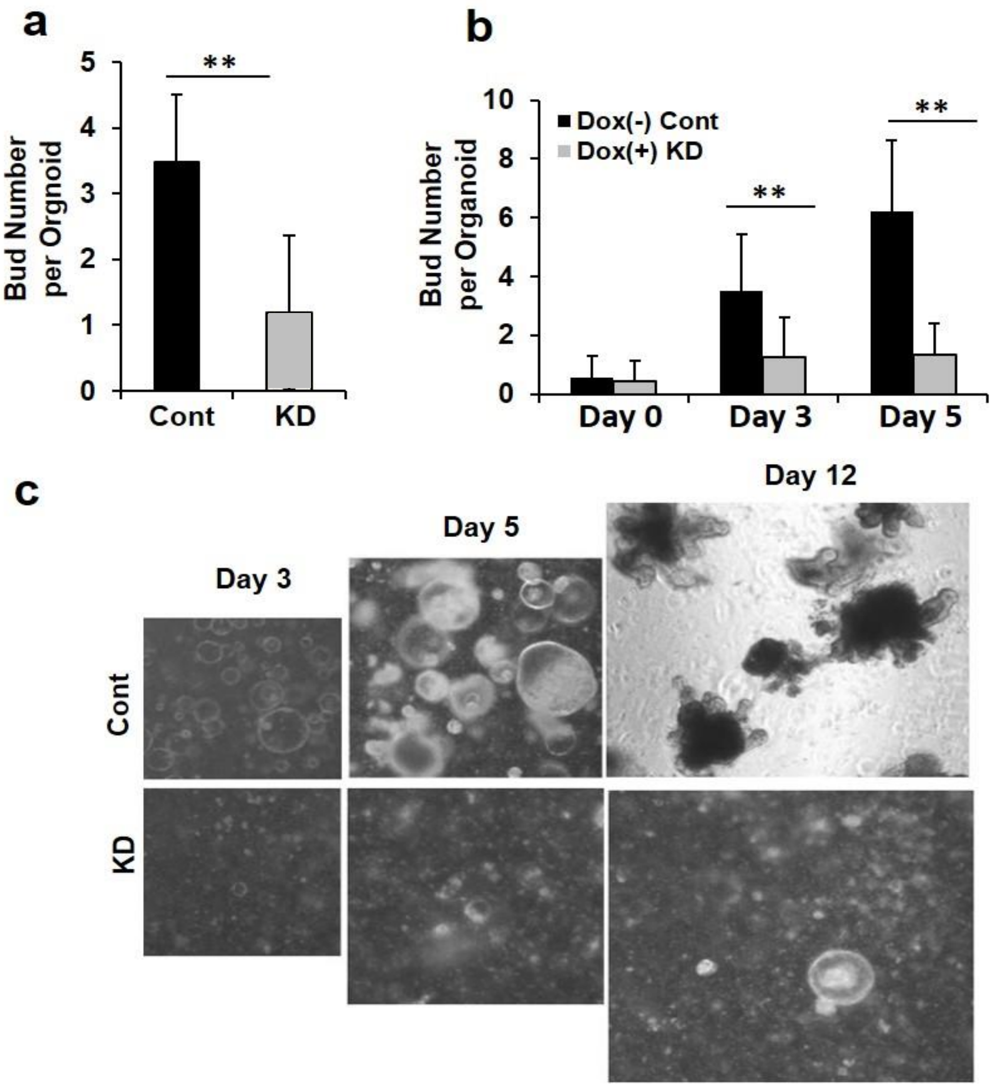

Supplementary Figure 5. Silencing *Hmga1* in organoids disrupts 3D organoid derivation and bud formation.

(a) Bud number is decreased in WT organoids transduced with lentivirus encoding shRNA targeting *Hmga1* (knock-down, denoted KD) as compared to WT organoids transduced with control virus (Cont) at day 10 following transduction. **P*<0.05 (student's t-test, n = 21/group; error bars represent standard deviations)

(b) Bud number is also decreased in organoids transduced with a lentiviral vector expressing inducible red fluorescent protein (RFP) and shRNA targeting *Hmga1*. Doxcyline (0.5µg/ml) was used to induce shRNA and RFP expression. **P*<0.05 (student's t-test, n = 20/group; error bars represent standard deviations)

(c) Crypt cells from *Hmga1* transgenics have decreased ability to generate 3D organoids and buds following transduction with lentivirus encoding shRNA targeting *Hmga1* (KD; bottom panels) compared to control lentivirus (Cont; top panels; 4X magnification).

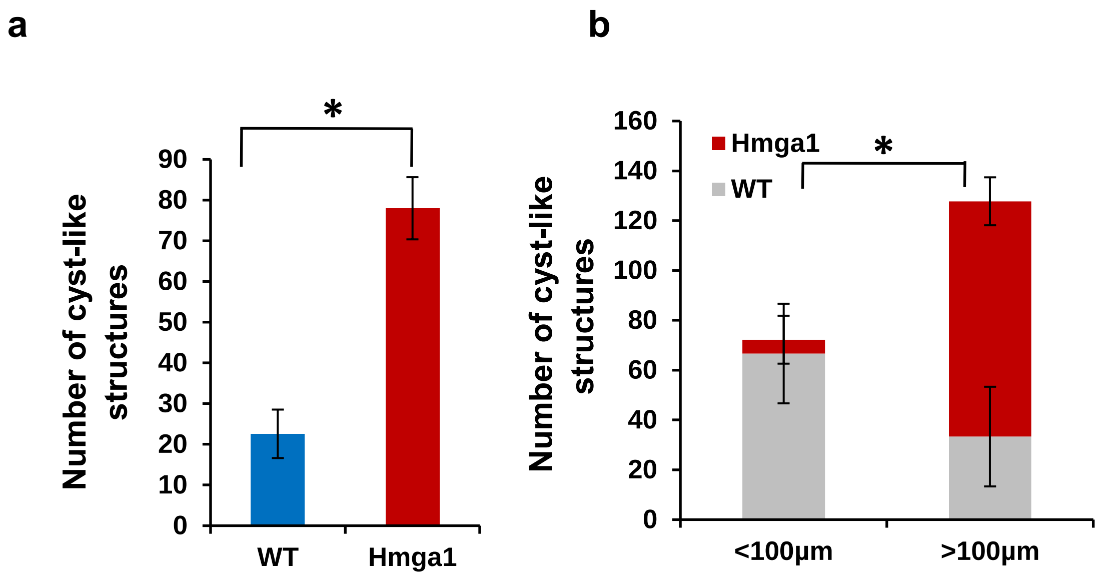

Supplementary Figure 6. *Hmga1* transgenic mouse organoids exhibit enhanced sensitivity to Wnt.
(a) Increased number of cyst-like structures form from *Hmga1* transgenic mouse organoids after exposure to Wnt3a (10 µ=ng/ml) as compared to WT organoids. *P<0.05 (student's t-test; error bars represent standard deviation)
(b) Greater diameter of cyst-like structures from *Hmga1* transgenic mouse organoids form after exposure to Wnt3a (10 ng/ml) as compared to WT organoids. *P<0.05 (student's t-test; error bars represent standard deviation)

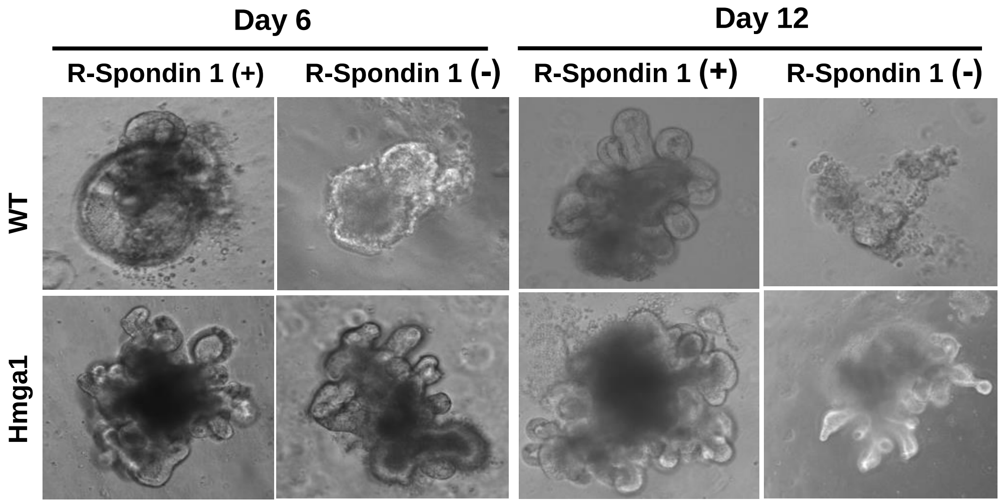

Supplementary Figure 7. *Hmga1* organoids remain viable in the absence of R-Spondin 1 (R-Spo1) for up to 12 days. Representative images of WT and *Hmga1* organoids cultured with or without R-Spo1 (1µg/ml) at 6 and 12 days.

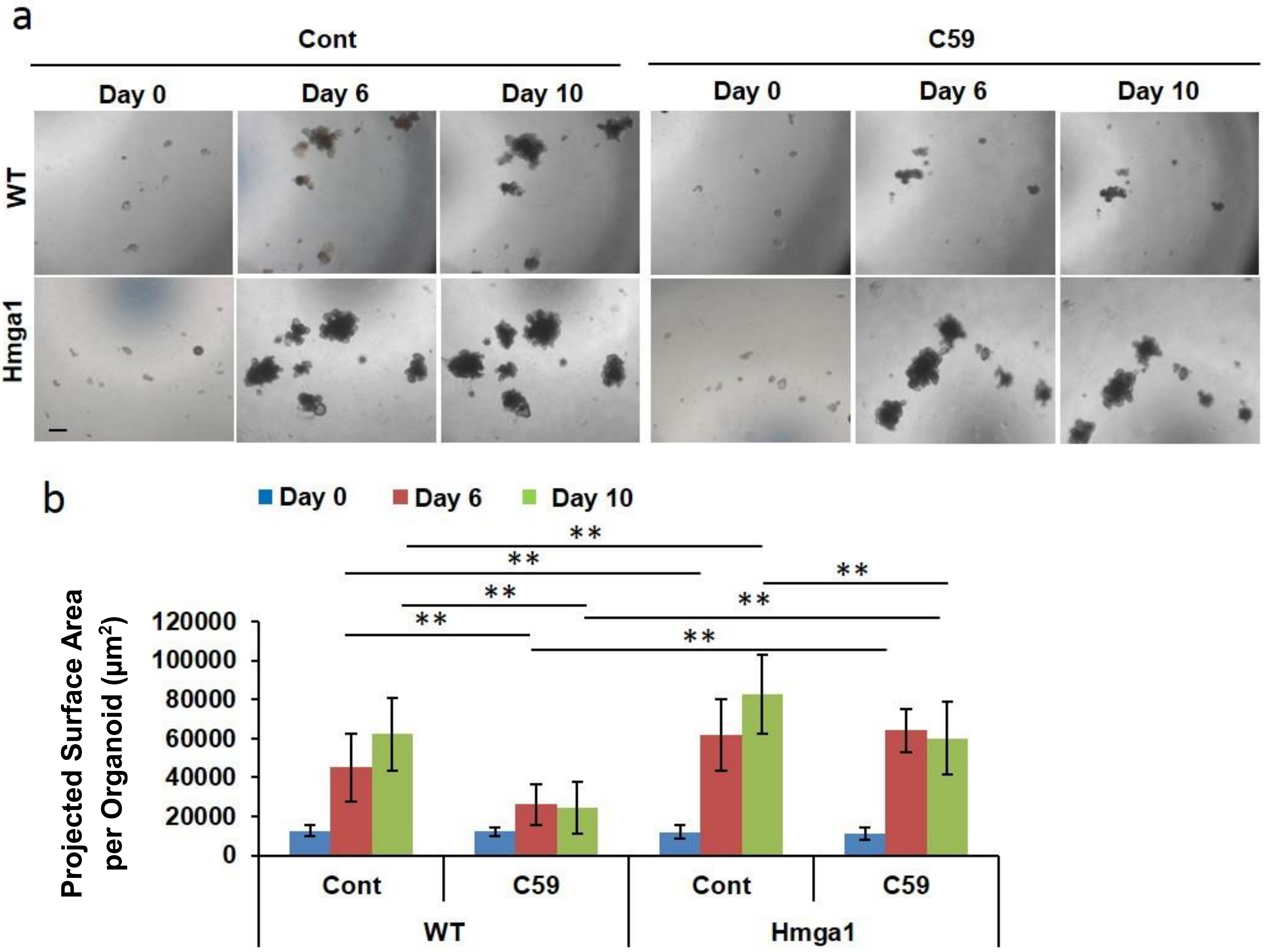

Supplementary Figure 8. *Hmga1* organoids are relatively resistant to the Wnt inhibitor C59.
(a) Crypt cells isolated from WT or *Hmga1* transgenic mouse small intestinal epithelium were selected for similar size and small cell numbers by passage through a 40 µm filter. Crypts were cultured with control vehicle alone (DMSO) or the Wnt inhibitor C59 (0.25 µM) for 10 days. Images were taken at days 0, 6, and 10 following crypt isolation and placement in 3D culture.
(b) Projected surface area per organoid was calculated using phase microscopy (Image Pro-plus 6). WT crypts cultured in the presence of C59 have a significantly smaller projected surface area at days 6 and 10 as compared to WT crypts cultured under control culture conditions at the same time points. As before, the *Hmga1* crypts generate organoids with a greater projected surface area as compared to WT organoids when cultured under control conditions. Unlike the WT organoids, the *Hmga1* organoids are relatively resistant to C59. Projected surface area was similar in *Hmga1* organoids cultured under control conditions or with C59 at day 6, although the projected surface area decreased modestly in the *Hmga1* organoids at day 10 with C59 as compared to *Hmga1* organoids cultured under control conditions. ***P <0.01* (student's t-test; error bars represent standard deviations).

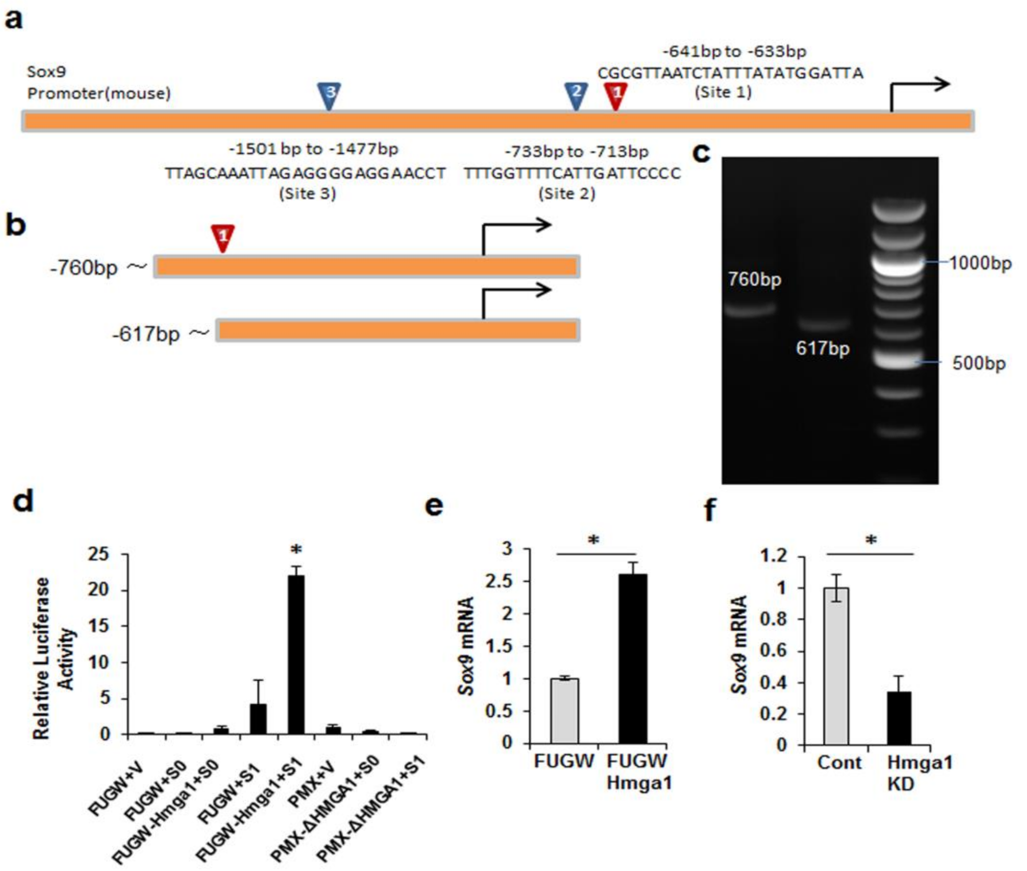

Supplementary Figure 9. Hmga1 directly induces the murine *Sox9* promoter

(a) Diagram of the murine *Sox9* promoter with Hmga1 DNA binding sites (triangles) predicted by an *in silico* DNA binding site prediction algorithm. (MatInspector[60]). The black arrow indicates the transcription start site. The red triangle indicates the predicted DNA binding site (site 1) that was confirmed by chromatin immunoprecipitation (ChIP) and showed the greatest Hmga1 occupancy. The blue triangles indicate two additional predicted Hmga1 DNA binding sites; only site 2 was confirmed by ChIP; site 3 had no significant enrichment for Hmga1 binding.

(b) Diagram depicting the *Sox9* promoter sequence with the Hmga1 binding site 1(0～-760 bp) and the control construct lacking theHmga1 binding site 1 (0～-617 bp) as a control.

(c) PCR products of *Sox9* promoter sequences; the PCR products were cloned and sequenced to confirm that the *Sox9* promoter sequences had been amplified and cloned successfully.

(d) Hmga1 induces expression of the mouse *Sox9* promoter. HEK 293 cells were transduced and selected for stable expression of the empty luciferase vector (V), the *Sox9* promoter reporter construct lacking the Hmga1 Site 1 (S0: 0～-617 bp), or the *Sox9* promoter reporter construct with the Hmga1 Site 1 (S1: 0～-716 bp). Stable cell lines were then transduced with control lentiviral vector (FUGW) or lentiviral vector expressing *Hmga1* (FUGW-Hmga1). Hmga1 significantly induces expression of the *Sox9* promoter containing the Hmga1 binding Site 1, but not the empty luciferase vector nor the *Sox9* promoter construct lacking the *Sox9* Site 1. We also tested a dominant-negative HMGA1 protein which is not capable of binding to DNA (PMX-ΔHMGA1) or control vector (PMX) with the *Sox9* promoter constructs. Dominant-negative HMGA1 was not capable of activating *Sox9* promoter expression nor did it affect expression of the control vector lacking the Hmga1 Site 1 or empty vector. Experiments were performed in triplicate and repeated twice; error bars represent standard deviation; *P<0.05 (student's t-test.)

(e) *Sox9* mRNA expression is induced in WT organoids engineered to overexpress *Hmga1* (FUGW-Hmga1) as compared to WT organoids transduced with control lentiviral vector (FUGW). *P<0.05 (student's t-test; n=6/group, error bars represent standard deviation)

(f) Conversely, *Sox9* expression is repressed in WT organoids engineered with shRNA targeting *Hmga1* (KD) as compared to control shRNA (Cont). *P<0.05 (student's t-test; error bars represent standard deviation).

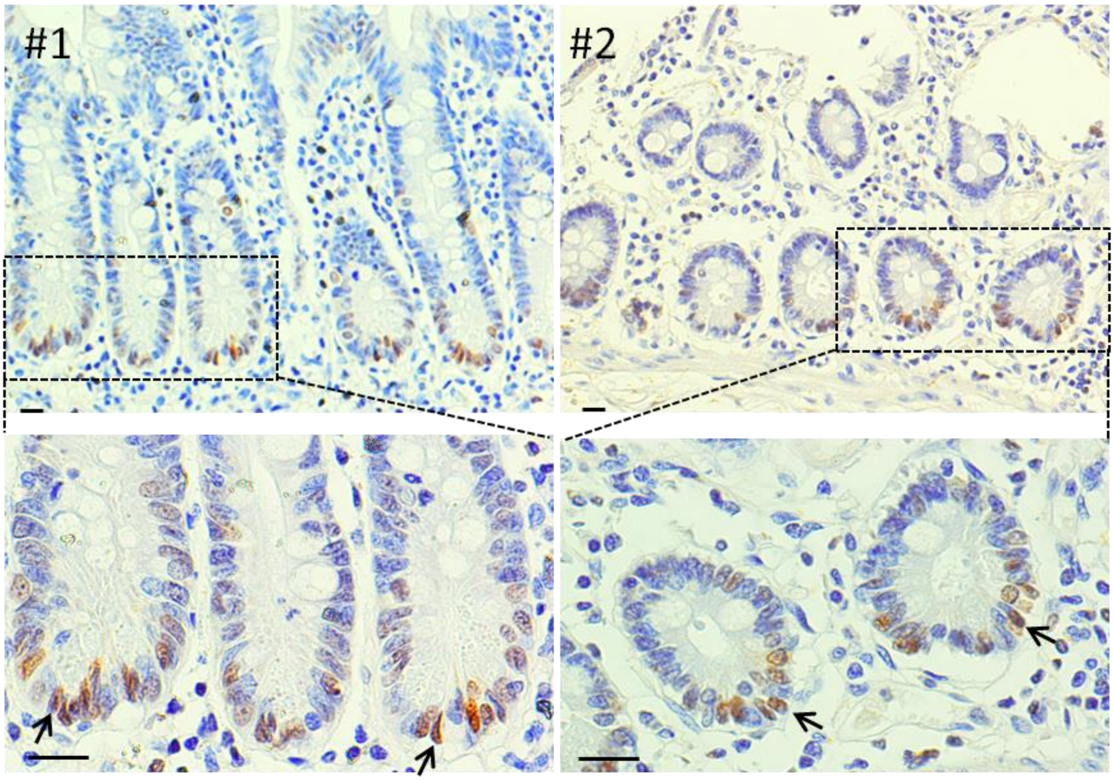

Supplementary Figure 10. HMGA1 protein in human small intestine localizes to the columnar cells at the base of the crypts. HMGA1 IHC stains brown and shows enhanced intranuclear staining (arrows) for HMGA1 at the crypt basal columnar cells. #1 and #2 denote samples from two individuals.

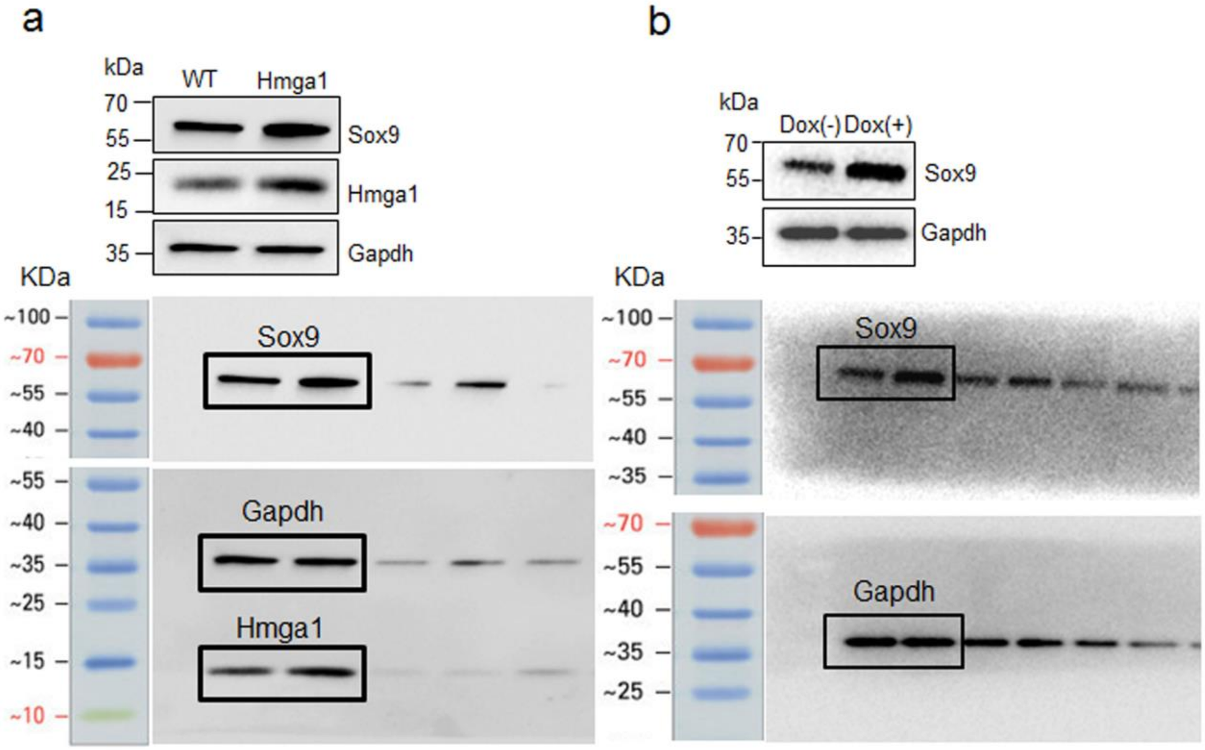

Supplementary Figure 11. Uncropped western blots imagines. ***(a)***Western blots for Fig. 6d. ***(b)*** Western blots for Fig. 7b.

Supplementary Table 1. Wnt receptor agonist and Wnt/Tcf4/β-catenin genes

Primer		Sequence
β-catenin	Forward	ATGGACGTGGGCGAACTTTTA
	Reverse	CGCCATCCCTGTCAATAATCTG
TCF4	Forward	CGAAAAGTTCCTCCGGGTTTG
	Reverse	CGTAGCCGGGCTGATTCAT
Fzd5	Forward	GGTGTGCCAGGAAATCACG
	Reverse	CACAAGCGGCCAGAATTGG
Fzd7	Forward	GCCACACGAACCAAGAGGAC
	Reverse	CGGGTGCGTACATAGAGCATAA
Lgr5	Forward	CCTACTCGAAGACTTACCCAGT
	Reverse	GCATTGGGGTGAATGATAGCA
Lrp5	Forward	AAGGGTGCTGTGTACTGGAC
	Reverse	AGAAGAGAACCTTACGGGACG
Lrp6	Forward	TTGTTGCTTTATGCAAACAGACG
	Reverse	GTTCGTTTAATGGCTTCTTCGC
Axin2	Forward	ATGAGTAGCGCCGTGTTAGTG
	Reverse	GGGCATAGGTTTGGTGGACT
Ephb2	Forward	GCGGCTACGACGAGAACAT
	Reverse	GGCTAAGTCAAAATCAGCCTCA
Ets2	Forward	CCTGTCGCCAACAGTTTTCG
	Reverse	TGGAGTGTCTGATCTTCACTGA
Prom1	Forward	GTTGAGACTGTGCCCATGAAA
	Reverse	GACGGGCTTGTCATAACAGGA
CD44	Forward	CACCATTGCCTCAACTGTGC
	Reverse	TTGTGGGCTCCTGAGTCTGA
Ascl2	Forward	AAGCACACCTTGACTGGTACG
	Reverse	AAGTGGACGTTTGCACCTTCA
Sox9	Forward	CAAGCGGAGGCCGAAGA
	Reverse	CAGCTTGCACGTCGGTTT
c-Myc	Forward	TTCTACGACTATGACTGCGGA
	Reverse	TGATGGAAGCATAATTCCTGCC

Supplementary Table 2. ChIP primers

Primers	Sequence
Sox9 site 1	**Forward - ACACCAGCTTCGTTGAACCAGAG -**<br>**Reverse - GGAAGCAAATGTTTGGGTGACTCA -**
Sox9 site 2	**Forward- ACTTGTCAGTTCAAGGTCGGCGTG -**<br>**Reverse - TGTGGTGACTGGAGCTTCTGCTG -**
Sox9 site 3	**Forward– CGAGCTTTTCAAAAGCATCCCAAAGA -**<br>**Reverse - TGATAAAGCGAATCGGCCTGTATC -**

Supplementary Table 3. Antibody list

Antigen	Clone/Cat#	Company	Applications
Anti-Hmga1	EPR7839/ab209761	Abcam	IHC (1:1000)<br>WB (1:1000)
Anti-Hmga1	Polyclonal/ab4078	Abcam	CHIP
Anti-Sox9	EPR14335/ab185230	Abcam	IHC (1:2000)<br>WB (1:1000)
Anti-lysozyme	EPR2994(2)/ab108508	Abcam	IHC, IF (1:1000)
Anti-GFP	Polyclonal/600—101-215	Rockland	IF (1:500)
B-Catenin	E247/ab32572	Abcam	IHC, IF (1:100)
EpCAM-FITC	G8.8/118210	Biolegend	IF (1:100)
Gapdh	Polyclonal/G9545	Sigma-Aldrich	WB (1:3000)

IHC: immunohistochemical analysis, ChIP: chromatin immunoprecipitation, IF: immunofluorescence, WB: Western blots
